# Neurons derived from individual early Alzheimer’s disease patients reflect their clinical vulnerability

**DOI:** 10.1093/braincomms/fcac267

**Published:** 2022-10-21

**Authors:** Bryan Ng, Helen A Rowland, Tina Wei, Kanisa Arunasalam, Emma Mee Hayes, Ivan Koychev, Anne Hedegaard, Elena M Ribe, Dennis Chan, Tharani Chessell, Dominic Ffytche, Roger N Gunn, Ece Kocagoncu, Jennifer Lawson, Paresh A Malhotra, Basil H Ridha, James B Rowe, Alan J Thomas, Giovanna Zamboni, Noel J Buckley, Zameel M Cader, Simon Lovestone, Richard Wade-Martins

**Affiliations:** Department of Physiology Anatomy and Genetics, University of Oxford, South Parks Road, Oxford OX1 3QU, UK; Kavli Institute for Nanoscience Discovery, University of Oxford, Dorothy Crowfoot Hodgkin Building, South Parks Road, Oxford OX1 3QU, UK; Kavli Institute for Nanoscience Discovery, University of Oxford, Dorothy Crowfoot Hodgkin Building, South Parks Road, Oxford OX1 3QU, UK; Department of Psychiatry, University of Oxford, Headington, Oxford OX3 7JX, UK; Kavli Institute for Nanoscience Discovery, University of Oxford, Dorothy Crowfoot Hodgkin Building, South Parks Road, Oxford OX1 3QU, UK; Nuffield Department of Clinical Neurosciences, University of Oxford, Dorothy Crowfoot Hodgkin Building, South Parks Road, Oxford OX1 3QU, UK; Nuffield Department of Clinical Neurosciences, University of Oxford, Dorothy Crowfoot Hodgkin Building, South Parks Road, Oxford OX1 3QU, UK; Nuffield Department of Clinical Neurosciences, University of Oxford, Dorothy Crowfoot Hodgkin Building, South Parks Road, Oxford OX1 3QU, UK; Department of Psychiatry, University of Oxford, Headington, Oxford OX3 7JX, UK; Department of Psychiatry, University of Oxford, Headington, Oxford OX3 7JX, UK; Department of Psychiatry, University of Oxford, Headington, Oxford OX3 7JX, UK; Department of Clinical Neurosciences, University of Cambridge, Cambridge CB2 0QQ, UK; Neuroscience, Innovative Medicines and Early Development, AstraZeneca AKB, Granta Park, Cambridge, CB21 6GH, UK; Department of Old Age Psychiatry, Institute of Psychiatry, Psychology and Neuroscience, Kings College London, London, SE5 8AF, UK; Invicro & Department of Brain Sciences, Burlington Danes Building, Imperial College London, Hammersmith Hospital, Du Cane Road, London, W12 0NN, UK; Medical Research Council Cognition and Brain Sciences Unit, Department of Clinical Neurosciences and Cambridge University Hospitals NHS Trust, University of Cambridge, Cambridge CB2 7EF, UK; Department of Psychiatry, University of Oxford, Headington, Oxford OX3 7JX, UK; Department of Brain Sciences, Imperial College London, Charing Cross Campus, London W6 8RP, UK; Dementia Research Centre, UCL Institute of Neurology, Queen Square, London, WC1N 3BG, UK; Medical Research Council Cognition and Brain Sciences Unit, Department of Clinical Neurosciences and Cambridge University Hospitals NHS Trust, University of Cambridge, Cambridge CB2 7EF, UK; Translational and Clinical Research Institute, Newcastle University, Newcastle, UK; Nuffield Department of Clinical Neurosciences, Headington, University of Oxford, Oxford OX3 9DS, UK; Kavli Institute for Nanoscience Discovery, University of Oxford, Dorothy Crowfoot Hodgkin Building, South Parks Road, Oxford OX1 3QU, UK; Department of Psychiatry, University of Oxford, Headington, Oxford OX3 7JX, UK; Kavli Institute for Nanoscience Discovery, University of Oxford, Dorothy Crowfoot Hodgkin Building, South Parks Road, Oxford OX1 3QU, UK; Nuffield Department of Clinical Neurosciences, University of Oxford, Dorothy Crowfoot Hodgkin Building, South Parks Road, Oxford OX1 3QU, UK; Department of Psychiatry, University of Oxford, Headington, Oxford OX3 7JX, UK; Janssen Medical UK, 50-100 Holmers Farm Way, High Wycombe HP12 4EG, UK; Department of Physiology Anatomy and Genetics, University of Oxford, South Parks Road, Oxford OX1 3QU, UK; Kavli Institute for Nanoscience Discovery, University of Oxford, Dorothy Crowfoot Hodgkin Building, South Parks Road, Oxford OX1 3QU, UK

**Keywords:** Alzheimer’s disease, induced pluripotent stem cells, clinical vulnerability, disease modelling, synapse loss

## Abstract

Establishing preclinical models of Alzheimer’s disease that predict clinical outcomes remains a critically important, yet to date not fully realized, goal. Models derived from human cells offer considerable advantages over non-human models, including the potential to reflect some of the inter-individual differences that are apparent in patients. Here we report an approach using induced pluripotent stem cell-derived cortical neurons from people with early symptomatic Alzheimer’s disease where we sought a match between individual disease characteristics in the cells with analogous characteristics in the people from whom they were derived. We show that the response to amyloid-β burden *in life,* as measured by cognitive decline and brain activity levels, varies between individuals and this vulnerability rating correlates with the individual cellular vulnerability to extrinsic amyloid-β *in vitro* as measured by synapse loss and function. Our findings indicate that patient-induced pluripotent stem cell-derived cortical neurons not only present key aspects of Alzheimer’s disease pathology but also reflect key aspects of the clinical phenotypes of the same patients. Cellular models that reflect an individual’s in-life clinical vulnerability thus represent a tractable method of Alzheimer’s disease modelling using clinical data in combination with cellular phenotypes.

## Teaser

Ng *et al.* report that Alzheimer’s disease patient-derived neurons recapitulate the clinical vulnerability to amyloid-β pathology of the same patients. Synapse loss in patient-derived neurons due to amyloid-β insult exhibits a spectrum of vulnerability *in vitro* that correlates with cognitive measures of the patients *in vivo*.

## Graphical Abstract

**Graphical Abstract:**
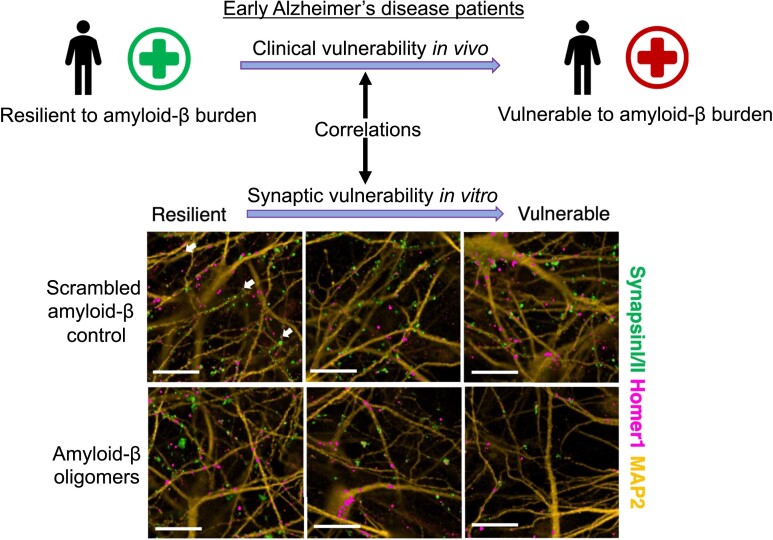

## Introduction

Alzheimer’s disease is the most common age-related neurodegenerative disease and cause of dementia, estimated to affect close to 50 million people in 2015 worldwide, with cases predicted to almost double every 20 years.^1^ Autosomal dominant mutations in the Amyloid Precursor Protein (*APP*) gene or genes encoding the APP proteolytic enzymes Presenilins 1 and 2 (*PSEN1, PSEN2*) are causative of early-onset familial Alzheimer’s disease. Largely based on insights from familial Alzheimer’s disease, alterations in amyloid-β (Aβ) generation, metabolism or clearance are thought to underlie the pathogenesis of late onset forms of sporadic Alzheimer’s disease. The wealth of evidence supporting this hypothesis has driven most drug development programmes to date. However, it is also apparent that whilst amyloid-related features predict clinical outcomes, this relationship shows very considerable inter-individual variation.^2^ Some individuals show evidence of extensive amyloid pathology yet little apparent clinical impairment and others have a relatively low amyloid burden in the context of moderately advanced dementia. Transgenic rodent models utilizing human familial Alzheimer’s disease gene mutations^3^ have been extensively used to model various aspects of APP/Aβ pathobiology but have not proved useful in exploring the mechanisms whereby this pathobiology affects disease pathogenesis and, as a consequence, we have no effective preclinical model of sporadic Alzheimer’s disease.

The advent of induced pluripotent stem cell (iPSC) technologies^4^ makes it possible to derive patient-specific cell lines capable of differentiating into various cell types and thereby human cellular models of disease. Although familial Alzheimer’s disease iPSC-derived cells exhibit pathological phenotypes *in vitro*, these are most obvious in APP-related phenotypes such as the production of an increased ratio of Aβ_1-42_ to Aβ_1-40_ peptides, whereas sporadic Alzheimer’s disease iPSC-derived cells typically do not share the same phenotypes.^5–7^ Recently, however iPSC-derived neurons were shown to display features *in vitro* that reflect analogous features from post-mortem materials from the same individuals—including, for example, quantitative measures of the generation of Aβ peptides.^8^ This has provided evidence of the feasibility of using individual cell models of disease to explore pathogenic mechanisms.

## Materials and methods

All reagents were purchased from Sigma-Aldrich unless stated otherwise. All iPSC-derived neuronal cultures were incubated at 37°C with 5% CO_2_.

### Deep and frequent phenotyping cohort pilot study and clinical data

The deep and frequent phenotyping (DFP) cohort pilot study protocol was previously published^9^ and a subset of the comprehensive clinical data and study participants (14 early symptomatic Alzheimer’s disease cases) was used for the analyses. These participants were recruited based on their clinical assessment meeting the criteria of early Alzheimer’s disease. The participant ages were in the age groups of 51–60 year (2/14), 61–70 years (4/14), 71–80 years (6/14) and 81–90 years (2/14) and 5/14 were female. One participant in the 51–60 years old age group with a family history of familial Alzheimer’s disease carried an *APP* mutation, while the remainder had a family history compatible with sporadic Alzheimer’s disease, hence no genetic tests for familial Alzheimer’s disease-linked mutations were offered in the clinical setting. Briefly for the study protocol, both amyloid PET imaging with [^18^F] AV45 (0–60 min, 150 ± 24 MBq) and magnetoencephalography (MEG) recordings were conducted once in 10/14 and 8/14 of the pilot study participants, respectively. The global efficiency metric from the γ-band (32–100 Hz) of the MEG raw data was used for analysis as it is the oscillation range linked to cognitive function and local connectivity.^10^ Lumbar puncture was performed over two visits 169 days apart in 12/14 of the study participants for CSF collection and Aβ_1-42_ peptide concentration was quantified via electrochemiluminescence in 96-well plates from meso scale discovery (Aβ peptide panel 1 with 6E10 antibody), before deriving the average values from the two visits for downstream analyses. All pilot study participants underwent a Mini Mental State Examination (MMSE; mean = 25.3) once and a MMSE score loss rate measuring cognitive decline was derived by dividing the MMSE score loss since estimated symptom onset (baseline MMSE score = 30) and the first visit to the clinic (MMSE score measurement) with the time since estimated symptom onset in days.

### Generation of Alzheimer’s disease patient-derived iPSC lines from blood samples

Blood samples (8 ml) were remixed via gentle inversion and centrifuged at 1800 g/20 min with brakeless deceleration. The plasma portion was removed, taking care not to disturb the whitish phase ring containing the peripheral blood mononuclear cells (PBMC). PBMC were diluted to 40 ml using phosphate-buffered saline (PBS; Thermo) (added dropwise to prevent osmotic shock) and centrifuged at 300 g/15 min. Cells were counted and plated at 5 × 10^6^/ml in Expansion I medium, which consists of StemSpan SFEM (Stem Cell Technologies) supplemented with lipid concentrate (1% Gibco), dexamethasone (1 µM), IGF-1 (40 ng/ml, R&D Systems), IL-3 (10 ng/ml, R&D Systems), EPO (2 U/ml, R&D Systems) and SCF (100 ng/ml R&D Systems). The remaining wells were filled with PBS to maintain a humid atmosphere (continued throughout all expansion and reprogramming steps). From DIV-1 to DIV-6, a 50% media change (Expansion I medium) was performed. Erythroblasts should appear ∼ DIV-5.

To purify the erythroblast population, 4 ml of Percoll was first added to a 15 ml tube. The wells were washed with DMEM (used for all washing steps) before a maximum of 8 ml of cell solution was slowly trickled onto the Percoll solution to collect erythroblasts. The solution was centrifuged at 1000 g/20 min with brakeless deceleration. The supernatant above the phase ring was transferred to a new tube and centrifuged at 300 g/5 min and washed twice to remove the Percoll. Purified erythroblasts were plated at 1–1.5 × 10^6^/ml in Expansion II medium, which has the same constituents as Expansion I medium except IL-3. On DIV-8/9, erythroblasts were collected, centrifuged at 200 g/5 min, re-suspended in Expansion II medium, and plated at 1–1.5 × 10^6^/ml to prevent cells differentiating down the erythroid lineage.

Before reprogramming erythroblasts to iPSCs, each well of a six-well plate was coated with 1 ml of 0.1% gelatine at 37°C for > 20 min. Mitomycin-C treated CF1 Mouse Embryonic Fibroblasts (MEF) were thawed and transferred to a tube containing 34 ml of MEF medium, which consists of Advanced DMEM supplemented with fetal calf serum (10%), GlutaMAX (1%) and 2-mercaptoethanol (0.1%), all purchased from Life Technologies. The gelatine was aspirated from the wells and 2 ml of MEF suspension were added per well. Plates were incubated overnight at 37°C before erythroblasts were plated after undergoing viral transduction.

Erythroblasts were collected and centrifuged at 200 g/5 min when they were ready to be infected with Sendai viruses expressing Yamanaka factors. The pellet was re-suspended in Expansion II media. 1.5 × 10^5^ cells were collected and made up to 200 *μ*l in Expansion II media. An aliquot from the CytoTune™-iPS 2.0 Sendai Reprogramming Kit (Thermo) was thawed on ice, mixed with 150 *μ*l of Expansion II media and added to the cell suspension. The entire suspension was transferred to a well in a 24-well plate. Viral supernatant was removed 23 h later by collecting cells and centrifuging at 300 g/4 min. The pellet was re-suspended in Expansion II media and transferred to a well in a 24-well plate before incubating for 48 h.

Finally, MEF medium was removed from feeder plates, which were washed with PBS before 1 ml of Expansion II media was added. The transduced erythroblasts were collected, centrifuged at 300 g/4 min, and re-suspended in Expansion II media. The cells were plated at a range of densities (1.5–4.5 × 10^4^/ml), which yielded ∼ 8–12 clones but allowed the clones to grow large enough for picking without overcrowding. A 50% media change was performed on the following days with the following media—DIV-5 (Expansion II media), DIV-7/8 (human embryonic stem cell medium which consists of KnockOut DMEM supplemented with 20% KnockOut serum replacement, 1% GlutaMAX, 1% non-essential amino acids, 0.1% 2-mercaptoethanol and 10 ng/ml BFGF), and DIV-10 (conditioned medium derived from MEF culture with human embryonic stem cell medium). Clones appeared ∼ DIV-15 and were picked ∼ DIV-22. If clones did not appear by DIV-40, the line was deemed to have failed to re-programme. Colonies that displayed embryonic stem cell-like morphology were selected via manual picking. All iPSC lines used in this study express the pluripotency markers Tra-1-60 and NANOG as measured by fluorescence-activated cell sorting.

### Maintaining iPSC culture and differentiation into iPSC-derived cortical neurons

iPSC cultures were maintained by growing the cells on Matrigel matrix (Corning) and feeding them with mTeSR^TM^ medium (STEMCELL technologies), which was replaced daily. We differentiated the iPSC lines into cortical neurons by overexpressing Neurogenin-2 (*Ngn2*).^11^ All 14 iPSC lines were transduced with lentivirus carrying the plasmids for a doxycycline-inducible system of *Ngn2* overexpression at a multiplicity of infection of four by the same co-author (A.H.) before the lines were distributed to all groups involved. We then plated the cells onto poly-ornithine (100 µg/ml) plus laminin (10 µg/ml) coated cell culture plates at 60 000 cells/cm^2^ (double for several lines which did not grow well) in mTeSR^TM^ medium (STEMCELL technologies) supplemented with Y-27632 (Tocris) at 10 µM on Day 0. The mTeSR^TM^ medium was replaced with Neurobasal^TM^ medium (Gibco) supplemented with B27^TM^ (Thermo), GlutaMAX^TM^ (Gibco), Penicillin-Streptomycin (Gibco), neurotrophin-3 (10 ng/ml), BDNF (10 ng/ml, Peprotech), doxycycline (1 µg/ml), laminin (200 ng/ml) and ascorbic acid (200 µM) five hours after plating. Subsequently, the cell culture medium was further supplemented with puromycin (1.5 µg/ml) on Day 2 only. To ensure *Ngn2* expression consistency within and across differentiation repeats, we included doxycycline in the neuronal media to maintain the *Ngn2* expression levels throughout the cortical neuron differentiation (Day 0 to 80 and during the exposure to Aβ).

The cells underwent the only and final passage on Day 4 with Accutase^TM^ and were plated at 25 000 cells/cm^2^ onto a confluent layer of rat cortical astrocytes (Thermo Fisher) in half-area 96-well plates. Rat cortical astrocytes were introduced to facilitate neuronal maturation,^12,13^ improve neuronal morphology for imaging assays, and improve cell attachment to withstand subsequent biochemical procedures. Half-feeding took place twice per week from Day 4 onwards with the abovementioned B27-containing medium. Finally, we also supplemented the medium with Ara-C (100 nM final concentration) on Days 10, 20, 40 and 60.

### Multi-electrode array (MEA)

The iPSCs were seeded directly onto the MEA plates, and 30 000 rat cortical astrocytes were seeded into each well of the MEA plates on Day 5 of the differentiation. From Day 45 onwards of the cortical neuron differentiation, 2 min long recordings were taken after 5 mins of plate settling time on the MEA reader regularly over time (Axion Biosystems, Maestro) with AxIS software v2.4.2.13 (Axion Biosystems). The plate was kept at 37°C while recordings were taken. The raw recording files were then extracted with AxIS software (Axion Biosystems) and processed with a custom script in MATLAB (version R2020b). Firing rate (FR) is defined as the number of extracellular electrical spikes in the millisecond window per recording length above noise (> 6 standard deviations). Burst rate (BR) is defined as the number of groups of a minimum five spikes with an inter-spike interval <100 ms counted per recording length.

### Alzheimer’s disease brain homogenate extraction

The extraction protocol of Alzheimer’s disease brain homogenate was modified from a published method.^14^ We sourced the post-mortem frozen frontal cortices from two Alzheimer’s disease patients (Patient #1: 73 years old, female, *APOE ε3/ε3*, Braak stage VI, 75 h post-mortem delay; Patient #2: 81 years old, male, *APOE ε4/ε4,* Braak stage VI, 26 h post-mortem delay) and a healthy control (70 years old, female, *APOE ε3/ε3*, Braak stage I, 81 h post-mortem delay) from the Oxford Brain Bank. We first thawed the brain tissues on ice prior to homogenization with Dounce homogenizers for 25 strokes in cold artificial CSF (aCSF: 124 mM NaCl, 2.8 mM KCl, 1.25 mM NaH_2_PO_4_ and 26 mM NaHCO_3_, pH = 7.4) with a ratio of 1 g of tissue to 4 ml of aCSF supplemented with a panel of protease inhibitors (5 mM EDTA, 1 mM EGTA, 5 ug/ml leupeptin, 5 µg/ml aprotinin, 2 µg/ml pepstatin, 120 µg/ml Pefabloc and 5 mM NaF). The homogenization was followed by centrifugation at 200 000 g for 110 min at 4°C and the supernatant was transferred into a Slide-A-Lyzer™ G2 Dialysis Cassette 2K MWCO in 100 times the volume of aCSF without protease inhibitors for 72 h. The aCSF was replaced every 24 h and the resultant aliquots were frozen at −80°C.

Three treatment controls were used in the synapse loss experiment using the Alzheimer’s disease brain homogenate, namely aCSF (vehicle), Aβ-immunodepleted Alzheimer’s disease brain homogenate and healthy brain homogenate. The Alzheimer’s disease brain homogenate aliquots underwent either Aβ immunodepletion or mock immunodepletion (the Alzheimer’s disease brain homogenate is used to cause synapse loss). Protein G agarose beads (Abcam) were washed three times in aCSF and centrifuged at 400 g for 5 min at 4°C. The beads were then re-suspended in a 50% slurry with aCSF. At the same time, the brain homogenate aliquots were thawed on ice and centrifuged at 16 000 g at 4°C for 2 min to remove any pellets. The agarose beads were then added to the brain homogenate at 3% v/v with 3 µg/ml of each 4G8 and 6E10 anti-Aβ antibodies (Biolegend) or an equal amount of normal mouse IgG antibodies (Abcam) added to the brain homogenate-agarose beads mixture. The mixture was left rotating at 4°C for 12 h before it was centrifuged at 400 g for 5 min at 4°C. The supernatant was transferred for another two rounds of mock or Aβ immunodepletion. Finally, the supernatant was mixed with Protein G agarose beads only at 2% v/v for 2 h before the brain homogenate was used for treatments. The healthy brain homogenate underwent mock immunodepletion in parallel in the same way as described.

The iPSC-derived neurons were incubated with either 25% Alzheimer’s disease brain homogenate (1:1 mixture of the two cortices) or aCSF without protease inhibitors as the treatment control in the cell culture medium (v/v) for 72 h at 37°C before paraformaldehyde fixation. Treatments with either Aβ-immunodepleted Alzheimer’s disease brain homogenate or healthy brain homogenate controls resulted in similar levels of synapse loss in the patient’s iPSC-derived cortical neurons to those treated with the Alzheimer’s disease brain homogenate (Supplementary Fig. 7C). This suggests that soluble factors other than Aβ itself in either the aged healthy brain or Alzheimer’s disease brain homogenates can also contribute to the synapse loss observed in this study.

### Meso-scale discovery immunoassay of Aβ peptides

For the quantification of Aβ peptides in cell supernatant, iPSC-derived neurons were grown as described previously without the Day 4 passage onto rat astrocytes until Day 40. Cell conditioned media was collected after 48 h and stored at −80°C. Cells were washed once with PBS, and M-PER™ (Thermo) added for 20 min on ice. Cell suspension was centrifuged at 14 000 g for 10 min at 4 ^o^C. The supernatant was collected, and protein concentration was quantified by a bicinchoninic acid assay (Thermo). Measurement of Aβ38, Aβ40 and Aβ42 was measured by electrochemiluminescence using the Meso Scale Discovery V-PLEX Aβ peptide panel (6E10), which was carried out according to the manufacturer’s protocol. Cell media samples were run in triplicate, with 25 µg of each cell lysate run in duplicate and kept on a plate shaker covered with a plate seal at room temperature during incubation. Aβ standards with a range of concentration levels were included for each immunoassay plate and the intra- and inter-plate percentages of coefficient of variation were <5% and approximately 10%, respectively, for all Aβ species (Supplementary Fig. 1). The Meso Scale Discovery Workbench 4.0 software was used to analyse Aβ levels. Conditioned media samples were normalized to the average total protein concentration in the lysate.

The Cisbio HTRF Aβ_1-40_ kit was used to quantify the levels of Aβ in the Alzheimer’s disease brain homogenates used in this study. The Alzheimer’s disease brain homogenate samples were diluted one to two, in the assay buffer for quantification. The Aβ levels were detected via homogeneous time-resolved fluorescence from a pair of antibodies. After mixing the kit reagents and the Alzheimer’s disease brain homogenate samples and leaving the plate to incubate overnight at 4°C, the Cisbio plate was read in a PHERAstar^®^ microplate reader (BMG Labtech) to detect fluorescence signals at wavelengths of 665 and 620 nm. The data were represented as ΔF, which is a relative value to the 665/620 nm signal ratio of the negative control. The absolute concentration was determined by fitting the signals to a standard curve using the provided Aβ_1-40_ calibrator. On average, 3255 pg/ml of Aβ_1-40_ was detected in the Alzheimer’s disease brain homogenate and the levels of Aβ_1-40_ were depleted/significantly lower in the Aβ immunodepleted samples and healthy brain homogenate (Supplementary Fig. 2). In brain homogenates prepared by the same protocol, we found the levels of Aβ_1-42_ in Alzheimer’s disease brain homogenate to be typically 211 pg/ml, compared with 5 pg/ml in Aβ-immunodepleted Alzheimer’s disease brain homogenate and 21 pg/ml in the healthy brain homogenate, as measured by the Meso Scale Discovery kit.

### Oligomerization of Aβ peptides and treatment in neuronal culture

Both lyophilized Aβ_1-42_ and treatment control scrambled Aβ_1-42_ peptides (Bachem, H-1368 and H-7406) were re-suspended to 1 mM in hexafluoro-2-propanol. The tubes were vortexed and left sitting at room temperature for 30 min, before they were aliquoted and dried in a Speed-Vac concentrator for 30 min. We kept the Aβ film at −80°C. To oligomerize the Aβ_1-42_ peptides, we first re-suspended the Aβ film in dimethyl sulfoxide to 5 mM before sonicating in water bath for 10 min. PBS was then added to the result in a 100 µM solution and the tubes were left stationary at 4°C for 24 h. Just before treating the cells with Aβ oligomers, the solution was centrifuged at 14 000 g for 10 min at 4°C to remove any precipitate/fibrils. Both Aβ_1-42_ and the scrambled Aβ_1-42_ control appeared to aggregate *in vitro* using this protocol (Supplementary Fig. 3). We concluded that the Aβ_1-42_-driven synapse loss observed in this study was due to the unique sequence of Aβ_1-42_ but not its aggregation status. The choice of a 10 µM exposure was determined by experimental optimization with iPSC-derived cortical neurons (Supplementary Fig. 4).

Both lyophilized Aβ_25-35_ and treatment control Aβ_35-25_ peptides (Bachem, H-1192 and H-2964) were re-suspended to 2 mg/ml in deionized water and vortexed before incubating at 37°C for 2 h for oligomerization. The vial was then aliquoted and frozen at −80°C.

The iPSC-derived neurons were incubated with Aβ oligomers for 24 h before paraformaldehyde fixation.

### Transmission electron microscopy

Aβ_1-42_ oligomer samples were first applied onto a carbon-coated 3 mm copper grid (TAAB), which underwent glow discharge for 20 s, by adding 10 µl of 100 µM samples. The samples were incubated for 2 min at room temperature on the copper grid before staining with 2% uranyl acetate for 10 s. The grid was washed once with water and stored at room temperature. Transmission electron microscopy images were acquired using a Tecnai 12 TEM microscope (120 kV) with a Gatan US1000 camera.

### Immunocytochemistry

Adherent neurons were fixed in 4% paraformaldehyde for 5 min, followed by treatment with 0.5% saponin in PBS for 20 min for permeabilization. To block the samples, we treated the plates with 10% normal goat serum with 0.01% tween-20 in PBS for 30 min. Primary antibodies were then left incubating with the samples at 4°C overnight with 1% normal goat serum and 0.01% tween-20, before washing with PBS for three times. Secondary antibodies were then applied to 1% normal goat serum and 0.01% tween-20 at room temperature for 1 h before washing for another four times. The primary antibodies we used were Guinea pig anti-Synapsin I/II (Synaptic Systems, 1:500), rabbit anti-HOMER1 (Synaptic Systems, 1:500), chicken anti-MAP2 (Abcam, 1:1000), mouse anti-human nuclear antigen (Abcam, 1:200), rabbit anti-CUX2 (Abcam, 1:200) and rat anti-CTIP2 (Abcam, 1:500). The secondary antibodies we used were Goat anti-guinea pig Dylight 488 (Abcam), goat anti-mouse Alexa Fluor 488, goat anti-rabbit Alexa Fluor 555, goat anti-chicken Alexa Fluor 555, goat anti-rabbit Alexa Fluor 647 and goat anti-rat Alexa Fluor 647 (Thermo) at 1:1000 dilution.

### High-content imaging and analysis

#### Synapse

The 96-well plates were imaged on the Perkin Elmer Opera Phenix high-content imager. We captured 15 images per well with a 43 × objective at +1 µm focus level with the binning value of 1. We then analysed the image with the Harmony software v4.9 from Perkin Elmer with a customized pipeline. The MAP2-positive neurites were identified with 0.5 overall thresholds as the region of interest and resized by expanding outwards by 5 px to cover synaptic signals, which lay slightly above the MAP2 signals. Both presynaptic (Synapsin I/II) and post-synaptic (HOMER1) signals were then identified with Method A of the ‘Find spots’ function with threshold values of 0.17 and 0.14, respectively. We also filtered away the spots that were larger than 100 px^2^. Finally, the synapses were ascertained by finding HOMER1 signals in the vicinity of Synapsin I/II signal regions, which had been resized by expanding outwards by 5 px. The absolute number of synapses was then normalized to the total MAP2-positive area to derive synaptic density, which was used for all downstream analyses. All the values of synaptic density downregulation due to the Aβ extrinsic insults were then normalized to the corresponding treatment controls i.e. Aβ_1-42_ normalized to scrambled Aβ_1-42_, Aβ_25-35_ normalized to Aβ_35-25_ (reversed) and Alzheimer’s disease brain homogenate normalized to aCSF.

#### Cortical markers

We captured 15 images at −1, 0 and +1 and µm focus levels per well with a 20 × objective and binning value of 2. We analysed the images on the same Harmony software by first identifying human nuclei among the co-culture with rat astrocytes and filtering away the nuclei with circularity less than 0.6. The percentage of cortical marker-positive cells was calculated by selecting the human nuclei with cortical marker mean signal intensity greater than a threshold, which was determined as the mean intensity across all human nuclei. Finally, we derived relative cortical marker expression by normalizing the percentage of cortical marker-positive neurons to the geometric mean across all 14 patient lines.

### Statistical analyses

All quantitative graphs and statistical analyses were performed in GraphPad Prism 9.2.0. We assumed normal distribution for the correlations with clinical data and chose parametric statistical analyses for the following reasons: (i) the percentage synapse loss parameter caused by each type of extrinsic Aβ insult in the correlation datasets were subjected to the D’Agostino-Pearson normality test (Aβ_1-42—_MMSE: *K^2^* = 1.80 and *P* = 0.41; Aβ_1-42—_MEG: *K^2^* = 2.56 and *P* = 0.28; Aβ_25-35—_MMSE: *K^2^* = 6.88 and *P* = 0.03; Aβ_25-35—_MEG: *K^2^* = 6.61 and *P* = 0.04; Alzheimer’s disease brain homogenate_—_MMSE: *K^2^* = 0.45 and *P* = 0.80; Alzheimer’s disease brain homogenate_—_MEG: *K^2^* = 0.47 and *P* = 0.79) using the Prism software. Both the synapse loss data caused by Aβ_1-42_ and Alzheimer’s disease brain homogenate pass the normality test by accepting the null hypothesis of normality, but not the Aβ_25-35_ synapse loss data which exhibit relatively more skewness towards greater synapse loss due to its higher toxicity level. However, the Aβ_25-35_ synapse loss data were determined to be significantly more likely (close to 100% probability) to be normal as opposed to lognormal when both normality and lognormality tests were considered; (ii) quantile-quantile plots of normality tests suggest that these datasets follow a normal distribution and (iii) the datasets were derived from a cohort of AD patients. For the correlation representations by simple linear regression line fittings, we reported Pearson’s coefficient of correlation and two-tailed *P*-values to indicate statistical significance. A two-tailed Welch’s *t*-test was used to compare between the most vulnerable and the most resilient groups of patient lines in the MEA experiment involving treatment with Aβ_1-42_ oligomers. A non-parametric one-way ANOVA test i.e. Kruskal–Wallis test, was used for comparisons amongst the patient lines for synaptic density, cortical marker expression levels, synapse loss and *APOE* genotypes as these datasets consist of three independent neuronal differentiation repeats per patient line. * *P* < 0.05, ** *P* < 0.01, *** *P* < 0.001 and **** *P* < 0.0001.

### Ethics statement

The DFP cohort study was approved by the London Central Research Ethics Committee, 14/LO/1467. The human iPSC lines used for this study were derived from human blood erythroblasts (National Health Service Research Ethics Committee: 10/H0505/71) and were derived as part of the Innovative Medicine Initiative-European Union sponsored Stem Cells for Biological Assays of Novel Drugs and Predictive Toxicology consortium. Informed consent was obtained from all donors.

## Results

### Generation of a panel of iPSC lines from a comprehensively-phenotyped cohort of early symptomatic Alzheimer’s disease patients

We set out to ask whether the heterogeneity of Alzheimer’s disease patients could be accurately reflected in iPSC models by comparing clinical outcomes *in vivo* with patient-derived neuronal phenotypes *in vitro*. We asked specifically whether clinical vulnerability to Aβ burden in the brain could be reflected by Aβ-induced cellular vulnerability in neurons derived from the same patients. In this study, we tapped into the comprehensive clinical datasets of the DFP pilot cohort^9^ (Supplementary Table 1), we generated thirteen sporadic Alzheimer’s disease iPSC lines and one familial Alzheimer’s disease iPSC line (Patient #5) carrying an autosomal dominant *APP* mutation, for use in our experiments (Supplementary Table 2 and Supplementary Fig. 5). Previously, the DFP study has highlighted the heterogeneity of the disease, as have many others showing, for example, a statistically significant correlation between amyloid burden measured by both PET and CSF measures and clinical outcomes and also a very considerable inter-individual variation in the impact of that amyloid pathology.^15^ This suggests a difference in vulnerability or resilience in the face of amyloid pathology that might reflect differences either in the hypothesized amyloid cascade or in factors that interact with that cascade. In either case, further understanding of such differences might yield insights to support therapeutic discovery. Here, we seek to investigate if the functional consequences in response to Aβ burden in the brains of Alzheimer’s disease patients (instead of the accumulation of Aβ pathology *per se*^8^) can be recapitulated *in vitro* using iPSC models derived from the same patients.

### Levels of secreted Aβ_1-42_ from patient iPSC-derived neurons reflect the levels of donor Csf Aβ_1-42_

**Figure 1:**
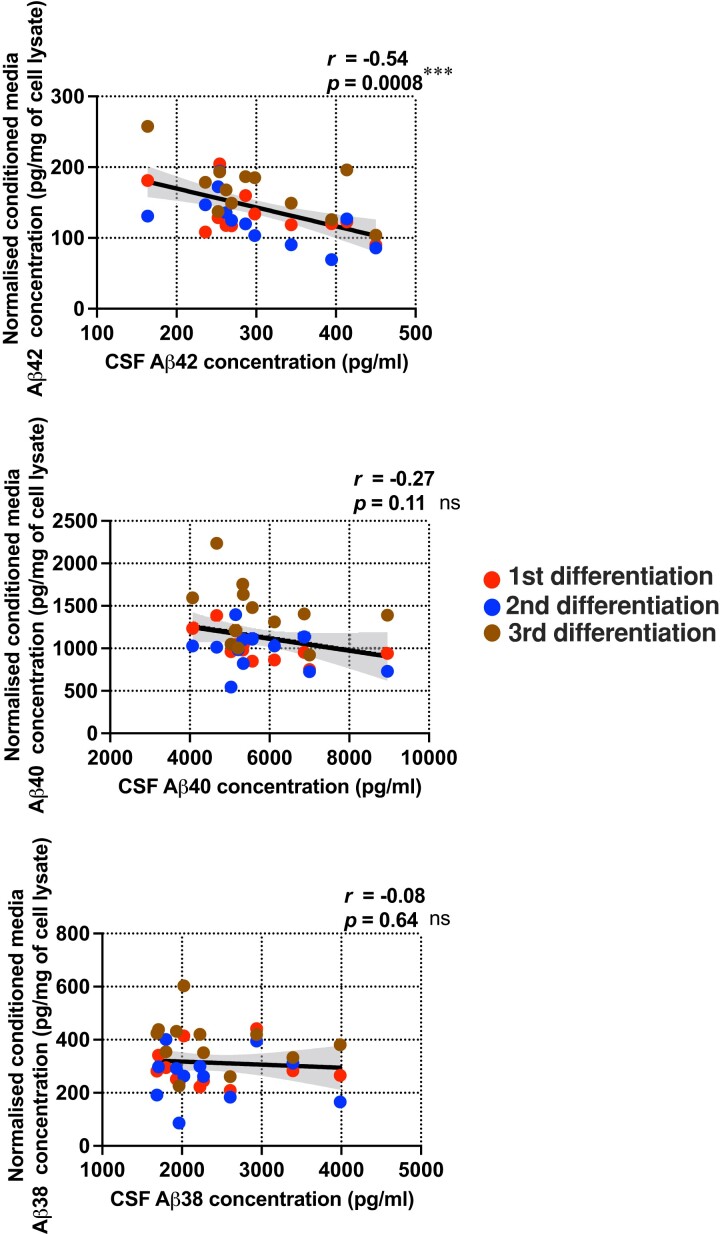
**Levels of secreted Aβ from Alzheimer’s disease patient iPSC-derived cortical neurons correlated with patient CSF Aβ levels.** Pairwise comparisons between the levels of secreted Aβ species from the patient-derived neurons and the levels of the same Aβ species in the patient’s CSF. Error band: 95% confidence interval (CI). There were *n* = 36 independent neuronal differentiation repeats per patient line. Pearson’s coefficient of correlation and its *P*-value were reported for statistical analysis.

To understand if patient-derived iPSC models recapitulate the in-life clinical measures of their donors, we first differentiated all 14 iPSC lines in parallel into cortical neurons in monoculture (Supplementary Fig. 6A) and showed that Aβ_1-42_ levels in the conditioned media correlate significantly and *negatively* with the same pathological Aβ species in the CSF from the patient donors (Fig. 1). In other words, patient-derived neurons that secreted greater levels of Aβ_1-42_ were generated from donors with lower CSF Aβ_1-42_ levels, a characteristic phenomenon of Alzheimer’s disease patients thought to be due to the sequestration of Aβ_1-42_ in non-soluble cortical amyloid plaques.^16^ Importantly, this relationship was not found for either Aβ_1-38_ or Aβ_1-40_ peptide comparisons and was not affected by the inclusion of the familial Alzheimer’s disease line. However, the Aβ_1-42_/Aβ_1-40_, and Aβ_1-38_/Aβ_1-40_ ratios were significantly increased in Patient #5 harbouring an *APP* mutation compared to the other patient lines, which consistent with the prior observation from another study.^17^ This result provides further evidence that patient-derived neurons reflect the pathological features *in vivo* of that patient. We next went on to examine patient-specific cellular vulnerability to Aβ *in vitro*.

### Patient iPSC-derived neurons demonstrate a spectrum of vulnerability to synapse loss after extrinsic Aβ insults *in vitro*

Dysregulation, and eventually loss of synapses, is one of the earliest pathological phenotypes of Alzheimer’s disease and leads to cognitive decline and memory loss.^18,19^ Neuronal activity measurement by MEG informs on synaptic dysregulation and loss and hence provides an opportunity to explore whether the individual impact of Alzheimer’s disease pathology on synaptic health in people *in vivo* is reflected in their cells *in vitro*. We, therefore, sought to investigate synapse loss in response to Aβ insults *in vitro;* iPSC lines were again differentiated in parallel into cortical neurons, this time plated in co-culture with rat cortical astrocytes (Supplementary Figs. 6A and B; Materials and methods). On Day 80 of the neuronal differentiation, we treated the neurons with a range of extrinsic Aβ insults, namely Aβ_1-42_ oligomers (10 μM), Aβ_25-35_ oligomers (20 μM) or human Alzheimer’s disease brain homogenate (25% v/v) and with scrambled Aβ_1-42_ peptides (10 μM), Aβ_35-25_ peptides (20 μM) or artificial CSF (aCSF; 25% v/v) as relevant treatment condition controls, respectively. These extrinsic Aβ insults were chosen due to their relevance to Alzheimer’s disease pathology: Aβ_1-42_ production is elevated in Alzheimer’s disease brain, and it is also the major pathological Aβ species found in amyloid plaques;^20,21^ Aβ_25-35_ is thought to represent the biologically active region of Aβ because it is the shortest fragment that exhibits large β-sheet aggregated structures and retains the toxicity of the full-length peptide;^22^ Alzheimer’s disease brain homogenate derived from post-mortem brain tissues consists of the composition of Aβ species that most closely recapitulates the pathological milieu. Further controls for the Alzheimer’s disease brain homogenate, including Aβ immunodepletion and a healthy brain homogenate, were performed as described in Materials and methods. We then performed immunocytochemistry on neurons with presynaptic (Synapsin I/II), post-synaptic (HOMER1: Homer Scaffold Protein 1) and dendritic (MAP2: Microtubule-associated Protein 2) markers before we conducted automated imaging on an Opera Phenix high-content confocal microscope.

**Figure 2:**
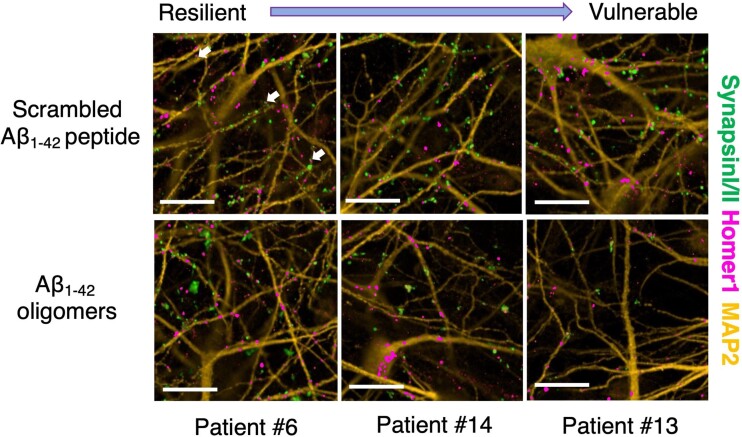
**Extrinsic Aβ insults resulted in a spectrum of vulnerability resulting in synapse loss in patient iPSC-derived cortical neurons.** Representative immunofluorescence images from three selected patient lines ranging from the least to the most vulnerable to Aβ_1-42_ oligomer insults relative to the scrambled peptide control treatment. The images are labelled with presynaptic (Synapsin I/II), post-synaptic (Homer1) and dendritic (MAP2) markers. White arrows indicate synapse examples with pre- and post-synaptic markers in apposition. Scale bar = 50 μm.

**Figure 3:**
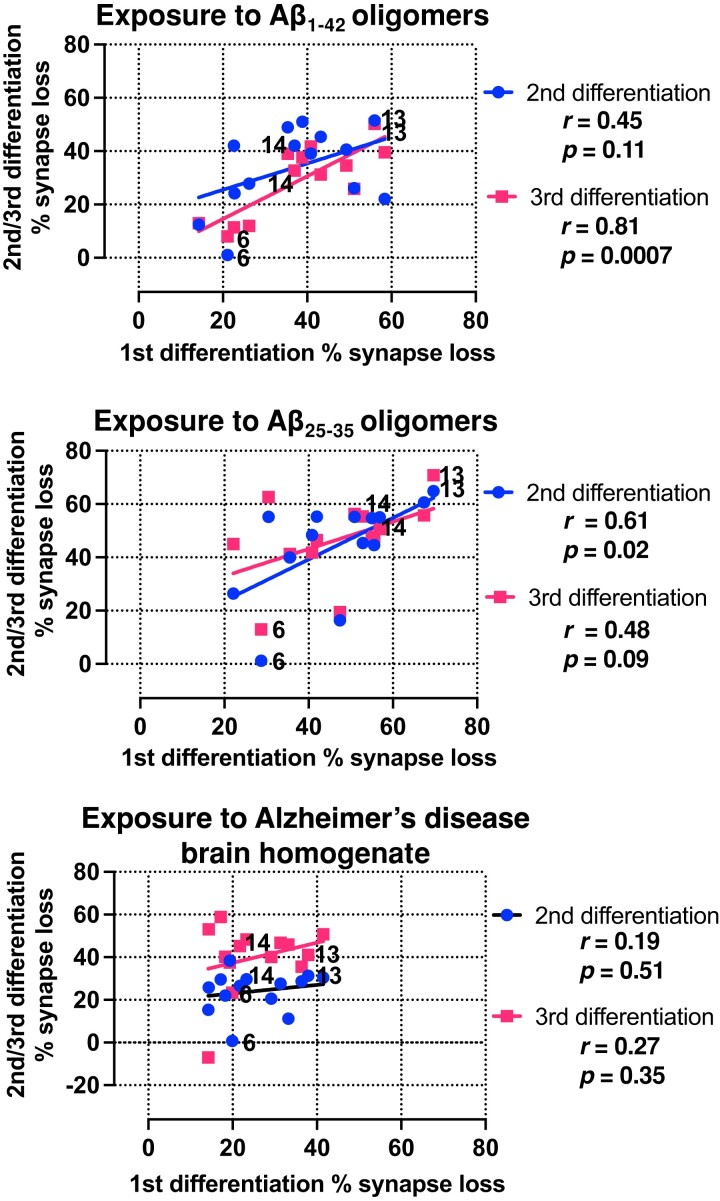
**Synaptic vulnerability to extrinsic Aβ insults in patient iPSC-derived cortical neurons remained consistent across neuronal differentiation repeats and types of Aβ insults.** Pairwise comparisons of the degrees of synapse loss between neuronal differentiation repeats caused by Aβ_1-42_, Aβ_25-35_ oligomers or Alzheimer’s disease brain homogenate. The same three selected patient lines from Fig. 2 are highlighted in the graphs. Pearson’s coefficient of correlation and its *P*-value were reported for statistical analysis.

All three exogenous Aβ treatments resulted in decreased synaptic density in all patient-derived cortical neurons relative to control treatments. However, the different patient lines showed different levels of impact of Aβ insults on synapse loss, allowing us to rank lines from the most resilient to the most vulnerable (Fig. 2 and Supplementary Figs. 6C and 7A). The exogenous Aβ treatment controls did not lead to synapse loss when compared with untreated iPSC-derived cortical neurons (i.e. neuronal media only) (Supplementary Fig. 7B). Notably, cellular vulnerability in the patient carrying the familial Alzheimer’s disease *APP* mutation (Patient #5), that generated the most endogenous Aβ_1-42_*in vitro* in Fig. 1, was within the range, but was relatively resilient to the impact of exogenous Aβ insults. All neurons displayed functional activity by firing action potentials on Day 80 of neuronal differentiation (Supplementary Fig. 6D). The synapse loss datasets demonstrated good reproducibility over three repeat independent iPSC differentiations. By comparing the extent of synapse loss between differentiation repeats, we confirmed that the specific levels of vulnerability in each line of iPSC-derived neurons in response to Aβ insults remained consistent across all differentiation repeats (Fig. 3 and Supplementary Fig. 8B). Importantly, similar patient line-specific vulnerability measured by synapse loss was also consistent across the different Aβ insults used, especially between Aβ_1-42_ and Aβ_25-35_ oligomers where there is a significant and positive correlation (Supplementary Fig. 8A). A positive correlation was also observed across differentiation repeats when the neurons were treated with Alzheimer’s disease brain homogenate (Fig. 3 and Supplementary Fig. 8B). The synapse loss data indicated that the degree of synapse loss due to the exposure to extrinsic Aβ in functional cortical neurons is patient-specific, cell-autonomous and reproducible across insults and differentiation repeats.

### Synaptic vulnerability to extrinsic Aβ insults in vitro reflects clinical vulnerability to Aβ burden *in vivo*

Next, we explored if the levels of synaptic vulnerability to Aβ insults in the patient-derived neurons *in vitro* were a reflection of the individual’s response to amyloid in life as measured using electrophysiological measures of synaptic activity and measures of cognitive decline, the ultimate clinical manifestation of synaptic dysfunction. Whilst in the *in vitro* experiments, the cells were exposed to the same amount of Aβ insult, but *in vivo* the individuals showed a range of amyloid burden. Global MEG recordings and cognitive decline measured by MMSE score loss rate (Supplementary Table 1) were therefore adjusted as a function of the patients’ individual levels of Aβ burden measured by amyloid PET standard uptake value ratios (SUVR) and CSF Aβ_1-42_ concentration, respectively. This yielded a personal ‘clinical vulnerability quotient’ representing the synaptic and cognitive response as a function of amyloid pathological load per individual. The resultant quotients were then rescaled within the DFP pilot cohort to range from zero (least vulnerable or most resilient), to one (most vulnerable, least resilient), thereby facilitating comparisons between the MMSE loss rate clinical vulnerability quotients and the MEG clinical vulnerability quotients.

**Figure 4:**
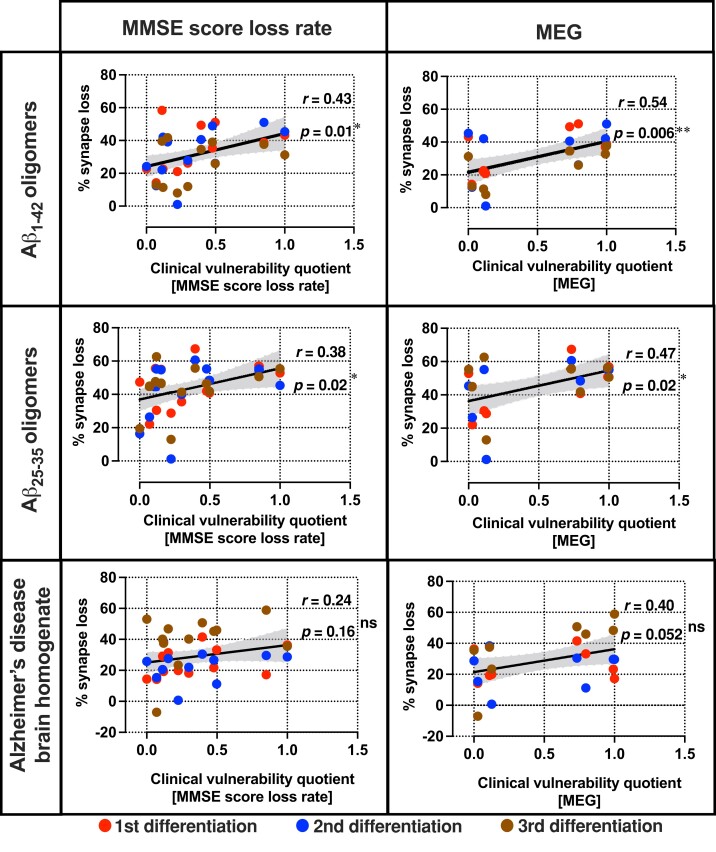
**Synapse loss due to Aβ insults *in vitro* reflects clinical vulnerability in the same patients to Aβ burden *in vivo*. Pairwise comparisons between the percentage of synapse loss and clinical vulnerability quotients.** Each row denotes the type of extrinsic Aβ insult used to induce synapse loss and each column denotes the selected clinical outcomes which have been corrected for Aβ_1-42_ concentration in the CSF (MMSE score loss rate) or amyloid PET SUVR (MEG). Error band: 95% confidence interval (CI). *n* = 35 (Aβ_1-42_—MMSE score loss rate), 36 (Aβ_25-35_ and Alzheimer’s disease brain homogenate—MMSE score loss rate) and 24 (all MEG) independent neuronal differentiation repeats per patient line. Pearson’s coefficient of correlation and its *P*-value were reported for statistical analysis.

Using this analysis, we found that the amount of synapse loss in patient-derived neurons caused by Aβ insults *in vitro* reflects the personal clinical vulnerability to Aβ burden *in vivo,* whether measured by the surrogate measure of synaptic number and function, MEG, or by cognitive decline, the core clinical manifestation of synaptic loss. Specifically, we observed a positive correlation between the percentage of synapse loss caused by both Aβ_1-42_ and Aβ_25-35_ oligomers and clinical vulnerability quotients, demonstrating that greater cellular vulnerability correlates significantly with greater clinical vulnerability in these patients (Fig. 4). Synapse loss due to exposure to human Alzheimer’s disease brain homogenate resulted in a similar correlation with clinical vulnerability quotients.

**Figure 5:**
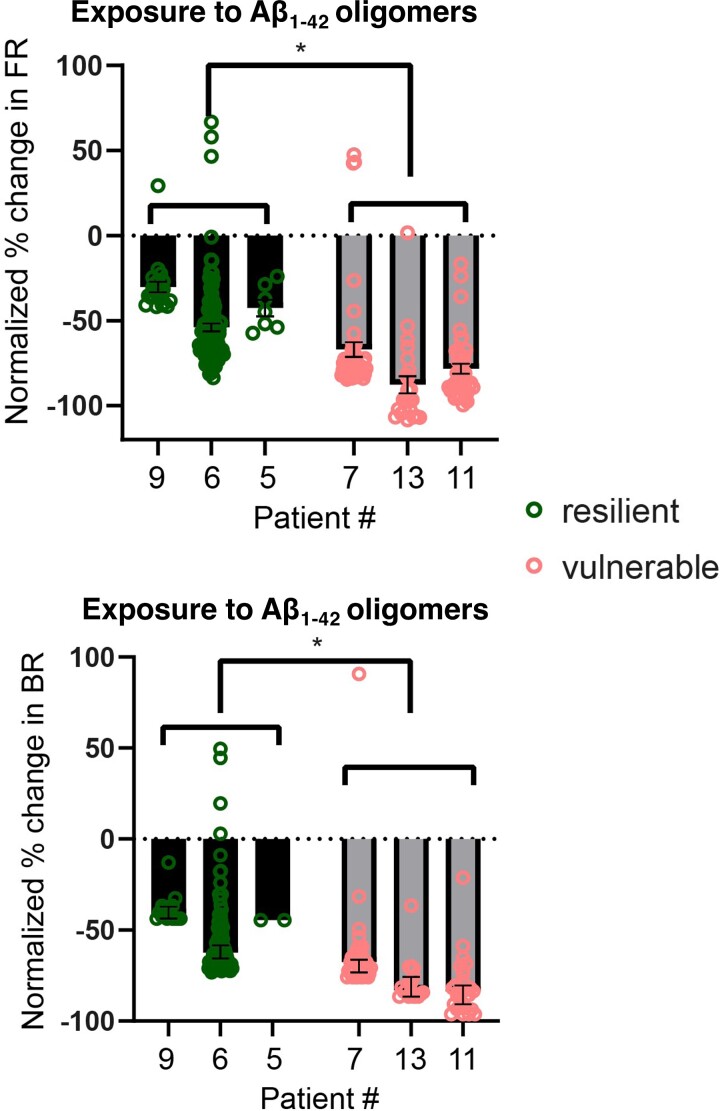
**Reductions in neuronal activity due to Aβ_1-42_ insults *in vitro* reflect synaptic vulnerability in the same patient lines.** Comparison of the resilient group (Patients #9, #6 and #5) and vulnerable group (Patients #7, #13 and #11) neuronal responses in their FR and BR to Aβ_1-42_ 10 μM on the second day of incubation. The vulnerable group showed a greater decrease in activity compared to the resilient group in both FR and BR. Each datapoint represents an electrode recording. *n* = 22 (#9), 114 (#6), 7 (#5), 49 (#7), 24 (#13) and 41 (#11) for the FR data, whereas *n* = 17 (#9), 93 (#6), 2 (#5), 43 (#7), 18 (#13) and 29 (#11) for the BR data. The percentage change from baseline was normalized against changes in untreated media control. Mean ± SEM; two-tailed Welch’s *t*-test was used for statistical analysis comparing the combined data of the resilient and vulnerable groups. *P* = 0.018 and *t* = 3.90 for the FR data and *P* = 0.016 and *t* = 4.11 for the BR data.

We then selected the three most vulnerable together with the three most resilient patient lines and investigated whether their electrophysiological activities were also differentially affected based on their synaptic vulnerability *in vitro*. As for the synaptic loss measures, the neurons derived from the most vulnerable patient lines exhibited greater reductions in firing and burst rates caused by the exposure to Aβ_1-42_ oligomers as compared to the most resilient patient lines (Fig. 5). The scrambled Aβ_1-42_ peptide control did not elicit any change in the levels of neuronal activity (Supplementary Fig. 9). Additionally, the differences in synapse loss in the patient-derived neurons could not be explained by their *APOE* variants (Supplementary Fig. 10) nor by the single case of an *APP* mutation carrier who scored as both relatively resilient to amyloid *in vivo* and to Aβ *in vitro,* suggesting that the resilience/vulnerability to Aβ is not driven either by the most significant genetic variant associated with sporadic Alzheimer’s disease or by mutations in the *APP* gene itself.

In conclusion, we show that neurons derived from Alzheimer’s disease patients retain the individual vulnerability to Aβ of the person from whom they were derived, demonstrated using both biomarkers and clinical measures that reflect the synaptic phenotypes measured *in vitro*.

## Discussion

In this study, we demonstrate for the first time that cellular vulnerability to Aβ insults *in vitro* reflects clinical vulnerability to Aβ burden *in vivo,* specifically in people living with Alzheimer’s disease dementia, by establishing the correlation between synapse loss in individual Alzheimer’s disease patient-derived neurons and their clinical outcomes. This was further supported by neurons from the more vulnerable group of patients exhibiting more deleterious responses to extrinsic Aβ insults as measured by their levels of neuronal activity as compared to the resilient group. This approach of integrating clinical in-life data with disease modelling in the laboratory presents a tractable method of Alzheimer’s disease modelling with iPSCs.

A decline in cognition estimated from a time since onset and current cognitive score, and ‘brain activity’ assessed using MEG were selected as clinical outcomes likely to be reflections of synaptic health and so broadly analogous to the synaptic loss data we measured *in vitro*. In both cases, we establish an individualized clinical outcome as a function of ‘amyloid burden’ using CSF Aβ_1-42_ and amyloid PET as measures of that burden. We report here that the amount of cognitive decline as a function of amyloid burden correlates with more severe Aβ-driven synapse loss and loss of synaptic function, as measured using MEA electrophysiology, in the patient-derived neurons. Although it has been known that synapse loss correlates with cognitive decline in Alzheimer’s disease,^19,23^ and that MEG identifies neurophysiological changes that are specific to Alzheimer’s disease, it remains unclear how different brain MEG signals change at different stages of Alzheimer’s disease progression.^24,25^ Interestingly, we find a clear correlation between *greater* brain activity levels measured by MEG correlating with more severe Aβ-driven synapse loss in the patient-derived neurons. This apparently counterintuitive observation is in fact in line with a considerable amount of evidence for hyperexcitability in the early phases of Alzheimer’s disease. Neurons exhibit hyperactivation, particularly during the mild cognitive impairment stage before hypoactivation as the disease progresses,^26,27^ and hyperexcitability leading to seizure activity is increased in Alzheimer’s disease, perhaps as a function of amyloid-related pathology.^28^ Indeed, preclinical evidence suggests that such excitability and seizure activity might accelerate the progression of tau-related pathology and contribute to regional Aβ deposition and hence actually be a target for therapy.^29–31^ Our findings substantiate the role of hyperexcitability in early Alzheimer’s disease and provide a model with which to explore such therapeutic discovery.

It has recently been shown that several measures of secreted Aβ peptides in iPSC-derived cortical neurons from Alzheimer’s disease patients reflect the extent of Aβ neuropathology of their donors.^8^ We extend that work on post-mortem, end-of-life, neuropathological findings to in-life, early in disease, clinical measurements by showing that the levels of Aβ_1-42_ secreted from patient-derived neurons correlate with the levels of the same pathological Aβ species in the patient CSF samples (Fig. 1). However, we have now shown that not only is there a correlation between cellular phenotypes and analogous phenotypes in the post-mortem brain and in patients, but that the functional consequences of those phenotypes—the response to Aβ as well as the amount of Aβ—are preserved in the cells. The familial Alzheimer’s disease case lies within the cellular-clinical correlation range in vulnerability to Aβ and strengthens the correlation (Fig. 4 and Supplementary Fig. 1^1^). The neurons from the familial Alzheimer’s disease individual belong to one of the more resilient patient lines *in vitro* even though this individual has the greatest Aβ burden measured by amyloid PET within this cohort, further reinforcing our interpretation that the iPSC models specifically reflect the vulnerability to Aβ measured by clinical outcomes instead of the levels of Aβ accumulation in the brain. Previously, a case of extreme resilience or resistance to amyloid has been reported in a person with an autosomal dominant mutation causative of Alzheimer’s disease who remained free from dementia late in life despite evidence of very extensive amyloid deposition.^32^ Sequencing suggested her apparently complete resilience was due to possession of two copies of a rare variant in *APOE ε3* (‘Christchurch’). We now show that relative resilience to amyloid is also identifiable in life in sporadic Alzheimer’s disease and can be modelled in cells *in vitro*. This resilience is considerably more subtle than that in the case report noted above, but just as in that case, understanding the cause of this resilience/vulnerability might yield insights into the pathogenesis of Alzheimer’s disease.

Exogenous toxic challenges to iPSC-derived neuronal cultures have experimental limitations. Although the synthetic Aβ oligomers used are disease-relevant, supraphysiological concentrations are often necessary to result in robust phenotypes within the experimental timeframe^33–38^ which is significantly shorter than the protracted exposure time to Aβ during disease. Lower exogenous Aβ concentration closer to the physiological level can sometimes be used for more sensitive readouts such as electrophysiological (patch-clamp) or gene expression changes^39,40^ but are insufficient to cause synapse loss in our case (Supplementary Fig. 4). Repeated treatments with a lower concentration of Aβ (low nM range) over a much extended period of neuronal culture may represent an alternative to mimicking the pathological milieu in an Alzheimer’s brain, but such lengthy experimental design *in vitro* poses major logistical challenges. To extend the physiological relevance of this study, therefore, we included an Alzheimer’s disease brain homogenate insult in the synapse loss experiments and relevant control conditions as described in Methods. Notably, the Aβ peptides and Alzheimer’s disease brain homogenate all showed the correlation between synapse loss *in vitro* and clinical vulnerability *in vivo*, although the use of synthetic Aβ oligomers generates more consistent and robust experimental data.

In conclusion, we reveal that cellular vulnerability reflects clinical vulnerability to Aβ in Alzheimer’s disease by modelling with patient iPSC-derived neurons and integrating cellular and clinical data from a highly phenotyped cohort. We first demonstrated the correlation between levels of Aβ_1-42_ secreted from patient iPSC-derived cortical neurons and the levels of the same pathological Aβ species in the patient CSF samples, and then we demonstrated Aβ-driven synapse loss and dysfunction in iPSC-derived neurons reflect relevant clinical outcomes as a function of Aβ burden in the brain. This work establishes the feasibility of modelling in-life Alzheimer’s disease clinical phenotypes with patient iPSC-derived neurons. Beyond that, as we can model inter-individual variability in clinical response to Aβ insult in an individual’s own iPSC-derived neurons *in vitro*, this raises the potential for interrogating mechanisms and identifying targets for precision therapy in human cell models.

## Supplementary Material

fcac267_Supplementary_Data

## Data Availability

The data that support the findings are presented in the figures or table included in the paper. The more detailed raw data of the experiments are available from the corresponding authors upon reasonable request. The data from the DFP cohort can be requested via the Dementias Platform UK online portal (https://www.dementiasplatform.uk/research-hub/data-portal).

## Abbreviations

Aβ =: amyloid-β
aCSF =: artificial CSF
BR =: burst rate
DFP =: deep and frequent phenotyping
FR =: firing rate
iPSC =: induced pluripotent stem cells
MEA =: multi-electrode array
MEF =: mouse embryonic fibroblast
MEG =: magnetoencephalography
MMSE =: mini mental state examination
PBMC =: peripheral blood mononuclear cell
PBS =: phosphate-buffered saline and
SUVR =: standard uptake value ratio
