# Supplementary material for: Neurons derived from individual early Alzheimer’s disease patients reflect their clinical vulnerability
Source: Brain Commun. 2022 Oct 21;4(6):fcac267. doi: 10.1093/braincomms/fcac267 (PMC9636855; doi:10.1093/braincomms/fcac267)
Supplement: fcac267_Supplementary_Data [file fcac267_supplementary_data.pdf]

Supplementary Table 1: Characteristics of the Deep and Frequent Phenotyping pilot cohort participants

			Alzheimer's disease biomarkers		Clinical outcomes		Clinical vulnerability quotients[§§§§]	
Patients	*MMSE score*	*APOE genotype*	*Cortical amyloid PET (SUVR)*	*CSF $A\beta_{1-42}$ concentration*[§] *(pg/ml)*	*MMSE score loss rate*[§§] *(per day)*	*Global MEG recording*[§§§]	*MMSE score loss rate quotients*	*MEG quotients*
1	22	ε4/ε4	1.60	298	1.5×10^{-3}	0.974	0.12	0.11
2	24	ε2/ε3	1.21	269	8.4×10^{-3}	0.983	0.85	1.00
3	23	ε4/ε4	1.28	344	6.3×10^{-3}	0.985	0.42	0.80
4	27	ε3/ε4	N/A	395	0.2×10^{-3}	N/A	0.00	N/A
5	26	ε3/ε3	1.63	254	0.8×10^{-3}	0.960	0.07	0.03
6	25	ε3/ε4	1.60	450	3.9×10^{-3}	0.979	0.22	0.13
7	22	ε3/ε3	1.28	252	3.7×10^{-3}	0.966	0.40	0.73
8	27	ε3/ε3	N/A	236	1.2×10^{-3}	N/A	0.15	N/A
9	29	ε3/ε4	N/A	262	3.0×10^{-3}	N/A	0.30	N/A
10	26	ε4/ε4	N/A	164	2.9×10^{-3}	N/A	0.48	N/A
11	24	ε3/ε4	1.30	414	1.9×10^{-3}	N/A	0.11	N/A
12	24	ε3/ε4	1.62	287	10.5×10^{-3}	0.944	1.00	0.00
13	26	ε3/ε4	1.56	N/A	3.1×10^{-3}	N/A	N/A	N/A
14	29	ε3/ε4	1.18	N/A	0.5×10^{-3}	0.961	N/A	0.99

[§] Average value between two visits which were 169 days apart

[§§] Derived from MMSE score loss since estimated symptom onset and the baseline visit when the participants underwent MMSE

[§§§] Global efficiency metric from the γ-band (32-100 Hz)

[§§§§] Derived by dividing clinical outcome measurements with corresponding Alzheimer's disease biomarkers (i.e., MMSE score loss rate/CSF $A\beta_{1-42}$ and MEG/amyloid PET) and then rescaled to range from 0 to 1 within the DFP pilot cohort

Note: N/A – not available

Supplementary Table 2: DFP pilot cohort patient-derived iPSC IDs	
**Patient #**	**iPSC ID**
1	BPC-927 03-01
2	BPC-928 03-07
3	BPC-929 03-07
4	BPC-932 03-03
5	BPC-933 03-12
6	BPC-934 03-02
7	BPC-936 03-07
8	BPC-937 03-01
9	BPC-939 03-01
10	BPC-940 03-08
11	BPC-943 03-03
12	BPC-944 03-04-01A
13	BPC-946 04-10
14	BPC-947 04-09

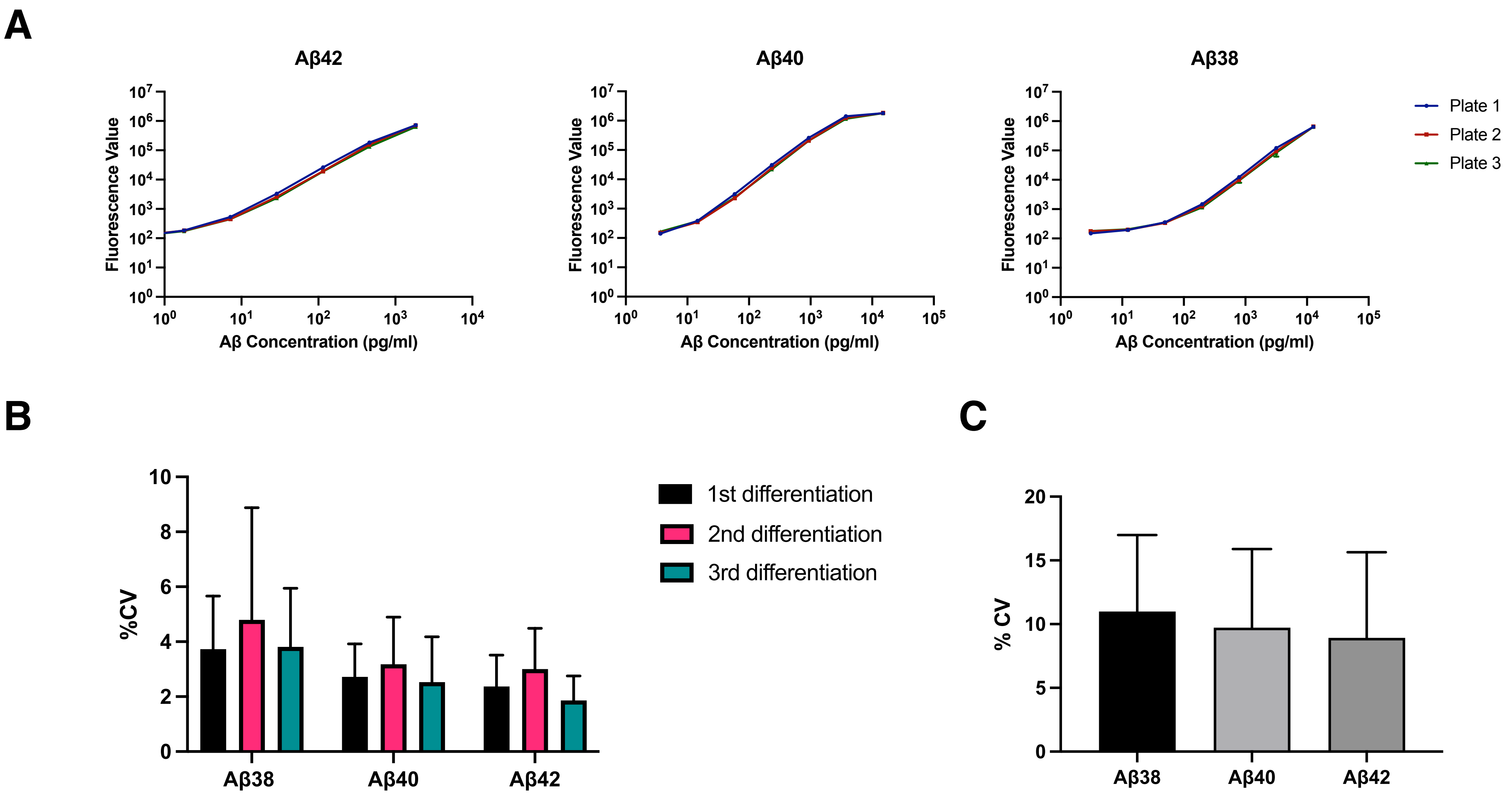

Supplementary Fig. 1

Supplementary Figure 1: Quality control of the quantification of secreted Aβ from the patient iPSC-derived cortical neurons using a Meso Scale Discovery immunoassay platform.

(A) Standard curves of each Aβ species for each immunoassay plate used, measured using the peptides with known concentration from the manufacturer. Mean ± SD. $n = 3$ technical repeats per plate.

(B) Bar graphs of the intra-plate percentage of coefficient of variation (%CV) for each neuronal differentiation repeat and for each Aβ species detected in the supernatant of the patient iPSC-derived cortical neurons. Mean ± SD. $n = 14$ patient lines per bar, with each datapoint an average of 3 technical repeats in the immunoassay plates.

(C) Bar graphs of the inter-plate %CV for each Aβ species of the standards across three plates. Mean ± SD. $n = 8$ Aβ standards with different concentration.

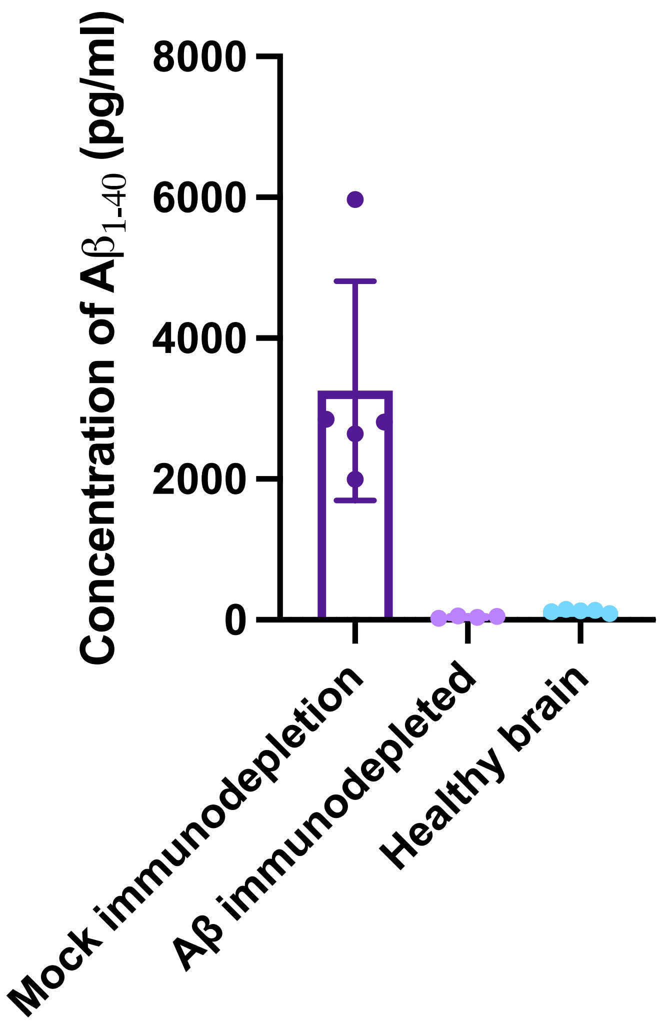

Supplementary Fig. 2

Supplementary Figure 2: Quantification of A$\beta_{1\text{-}40}$ in the brain homogenate samples.

AD brain homogenate derived from a post-mortem AD frontal cortex underwent mock immunodepletion while the same AD brain homogenate underwent Aβ immunodepletion in a separate sample using the 4G8 and 6E10 Aβ-targeting antibodies. Healthy brain homogenate derived from a post-mortem frontal cortex was also included in the analysis. n = 4 to 5 independent quantification repeats on the brain homogenate samples derived from the same frontal cortical tissues.

0 h 24 h

Scrambled $A\beta_{1-42}$

$A\beta_{1-42}$

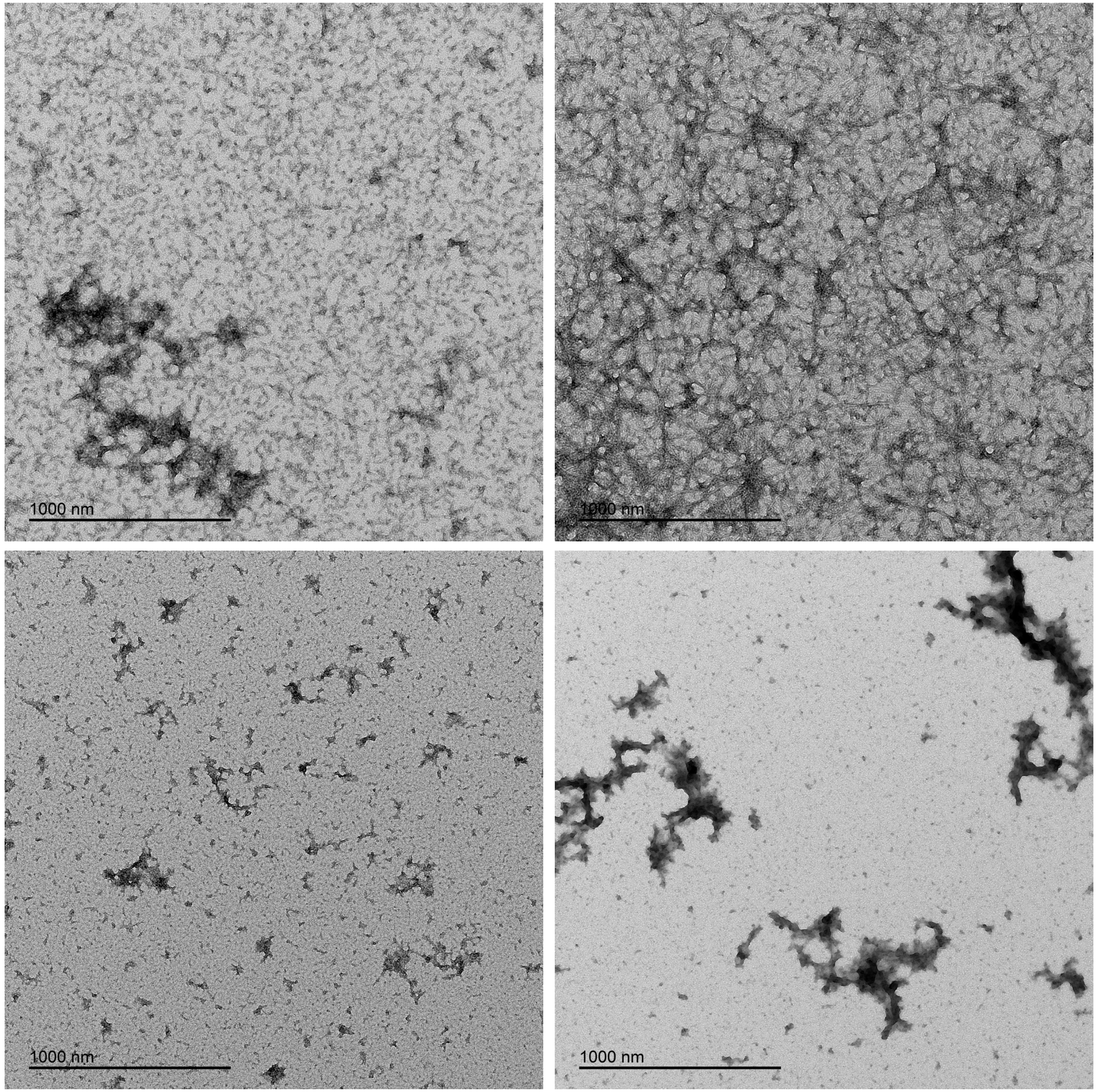

Supplementary Fig. 3

Supplementary Figure 3: Transmission electron microscopy images of scrambled $A\beta_{1-42}$ and $A\beta_{1-42}$ oligomerisation.

Both scrambled $A\beta_{1-42}$ and $A\beta_{1-42}$ peptide samples were imaged with an electron microscope at 0 h and 24 h post-incubation at 4 °C. Scale bar = 1 μm.

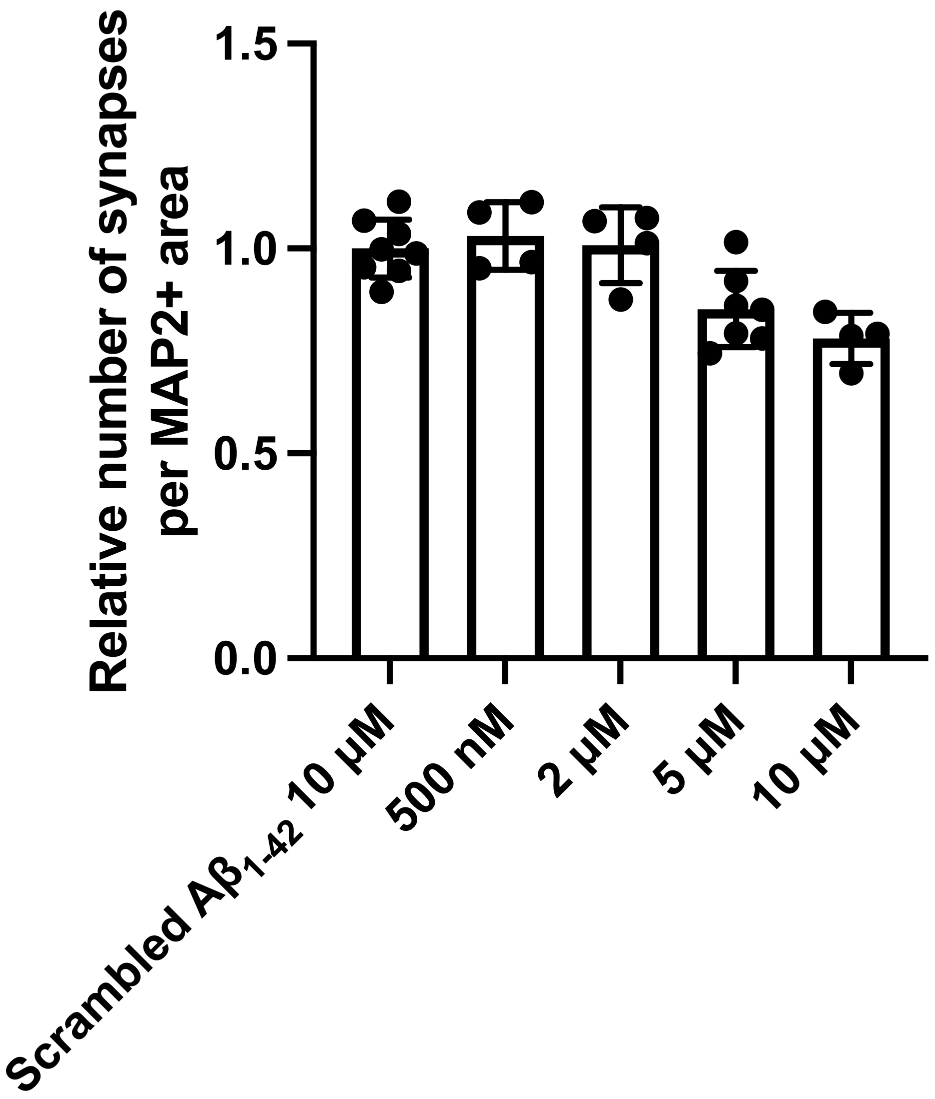

Supplementary Fig. 4

Supplementary Figure 4: Dose-dependent relationship of $A\beta_{1-42}$ oligomer-driven synapse loss.

Quantification of number of synapses in Day 89 iPSC-derived cortical neurons treated with various levels of $A\beta_{1-42}$ oligomers for 24 h relative to 10 μM scrambled $A\beta_{1-42}$ from one neuronal differentiation repeat. $n = 4$ to 8 replicate wells of neuronal populations.

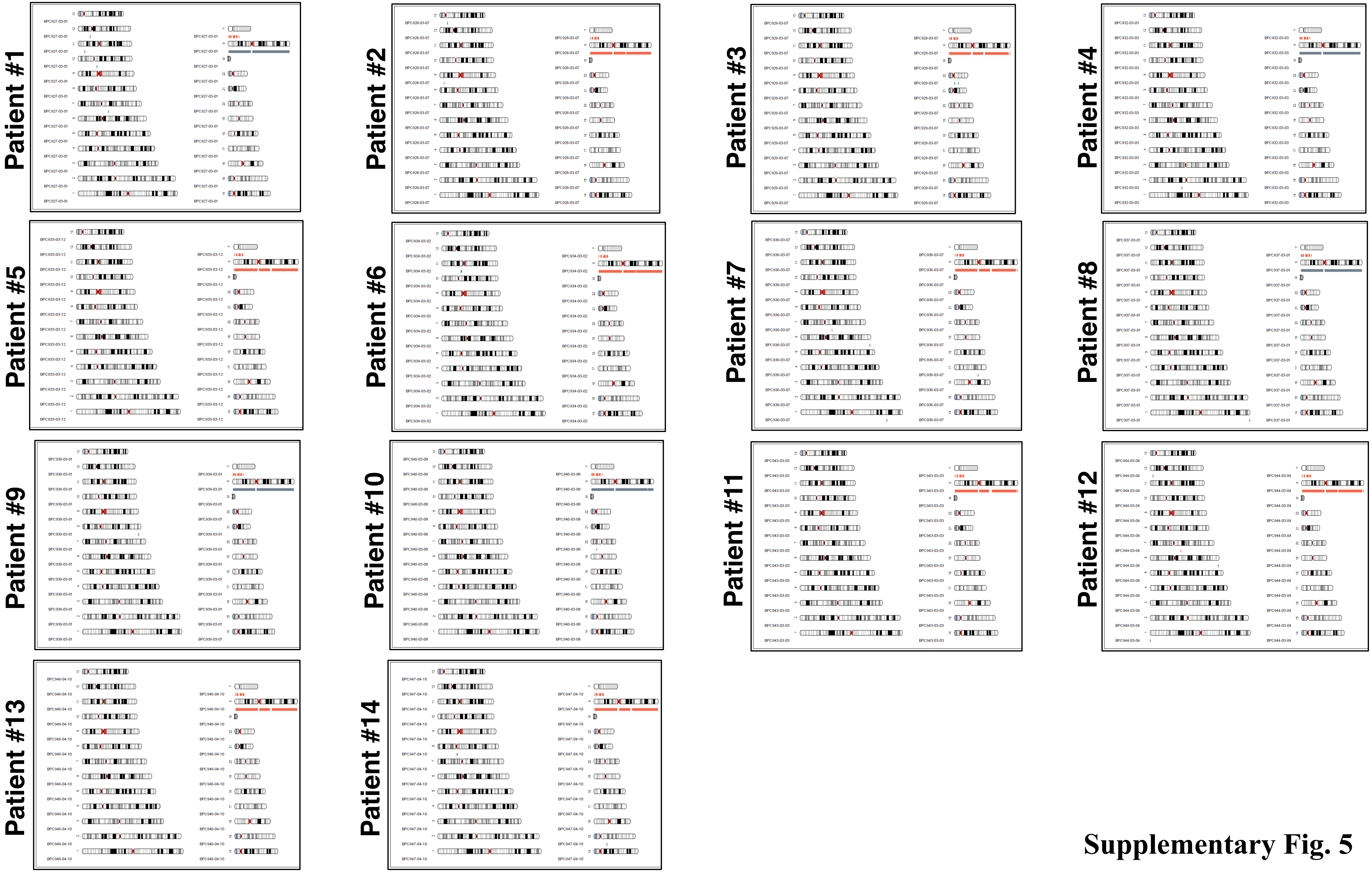

Supplementary Fig. 5

Supplementary Figure 5: Alzheimer's disease patient-derived iPSC quality controls.

Genome integrity of the Alzheimer's disease patient-derived iPSC lines, examined by the Illumina OmniExpress24 single nucleotide polymorphism array. Karyograms (KaryoStudio, Illumina) show amplifications (green)/deletions (orange)/loss of heterozygosity regions (grey) alongside the relevant chromosome. Female X chromosome is annotated in grey.

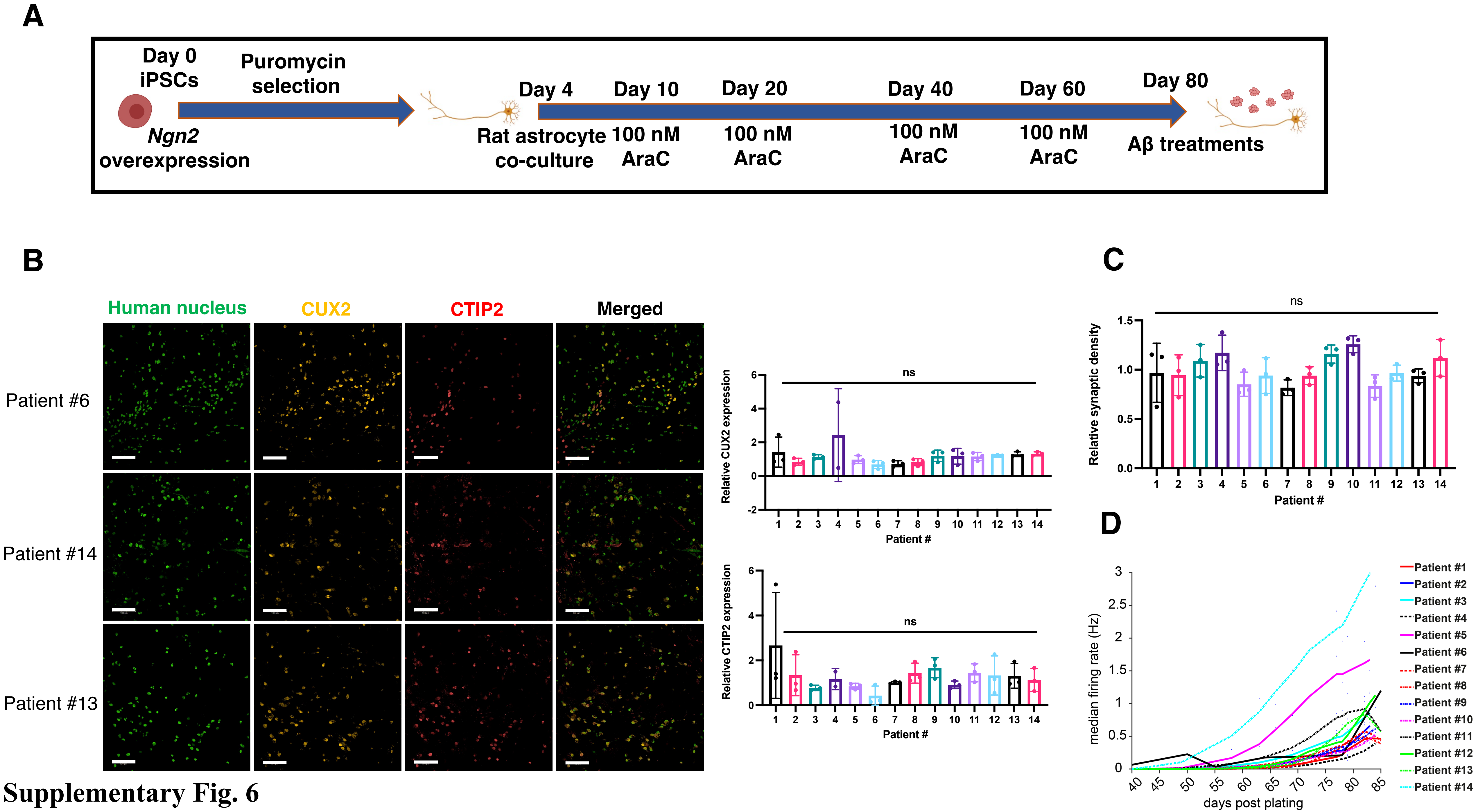

Supplementary Fig. 6

Supplementary Figure 6: Characterisation of iPSC-derived cortical neurons.

(A) Schematic of the cortical neuron differentiation protocol over 80 days.

(B) Representative images of cortical markers from three patient lines ranging from the least to the most vulnerable to Aβ insults, as well as the quantification of relative expression levels across all patient lines. Scale bar = 100 μm. Mean ± SD. *n* = 3 independent neuronal differentiation repeats. Kruskal-Wallis test was used for statistical analysis.

(C) Relative synaptic density across all patient-derived cortical neurons, normalised to the mean of synaptic density per neuronal differentiation. Mean ± SD. *n* = 3 independent neuronal differentiation repeats. Kruskal-Wallis test was used for statistical analysis.

(D) Neuronal activity increase over time measured by MEA from one neuronal differentiation. The figure plots smoothed line (the lowest function in MATLAB) of extracellular firing rate medians in Hz of cortical neurons between Day 40 to Day 85 post plating on the MEA plate. The small dots are the raw data points recorded. Each raw recording has the length of 2 min from which median was calculated.

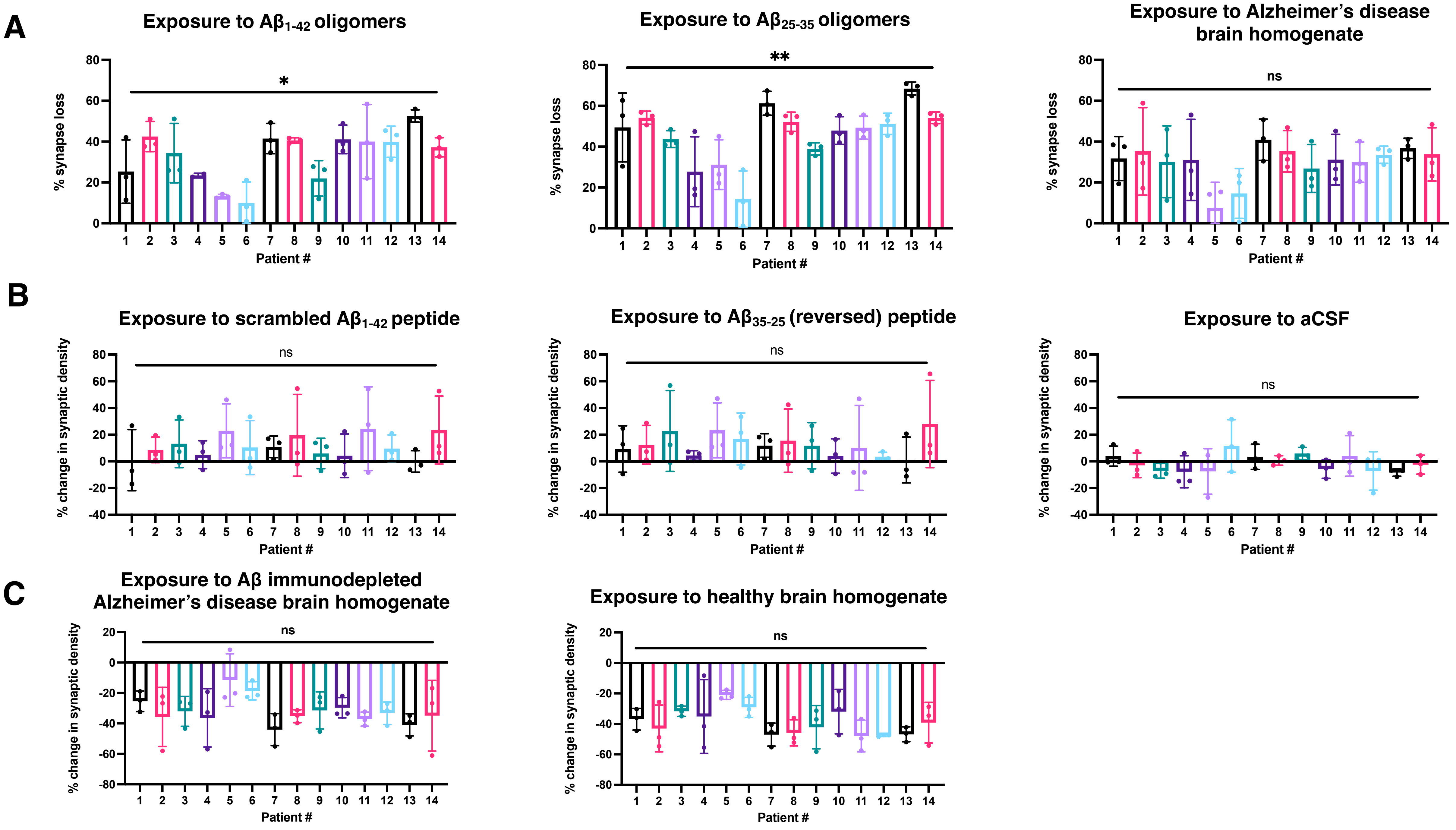

Supplementary Fig. 7

Supplementary Figure 7: Varying levels of synapse loss caused by Aβ insults.

(A) Percentage of synapse loss caused by 10 µM $Aβ_{1-42}$ oligomers, 20 µM $Aβ_{25-35}$ oligomers and 25% v/v Alzheimer's disease brain homogenate across all patient lines normalised to their respective treatment controls i.e. 10 µM scrambled $Aβ_{1-42}$ peptide, 20 µM $Aβ_{25-35}$ peptide and 25% v/v aCSF, respectively.

(B) Percentage change in synaptic density caused by 10 µM scrambled $Aβ_{1-42}$ peptide, 20 µM $Aβ_{25-35}$ peptide and 25% v/v aCSF across all patient lines normalised to the untreated group (i.e. neuronal media only).

(C) Percentage change in synaptic density caused by 25% v/v Aβ immunodepleted Alzheimer's disease brain homogenate and healthy brain homogenate across all patient lines normalised to the 25% v/v aCSF treatment group.

Mean ± SD. $n = 3$ independent neuronal differentiation repeats. Kruskal-Wallis test was used for statistical analysis. The y-axis in (A) is represented as "% synapse loss" as all three types of extrinsic Aβ insults resulted in synapse loss and the same readout is used for the correlation analyses in Figure 2A. The y-axes in (B) and (C) are represented as "% change in synaptic density" as they were intended to be treatment controls and did not necessarily result in synapse loss.

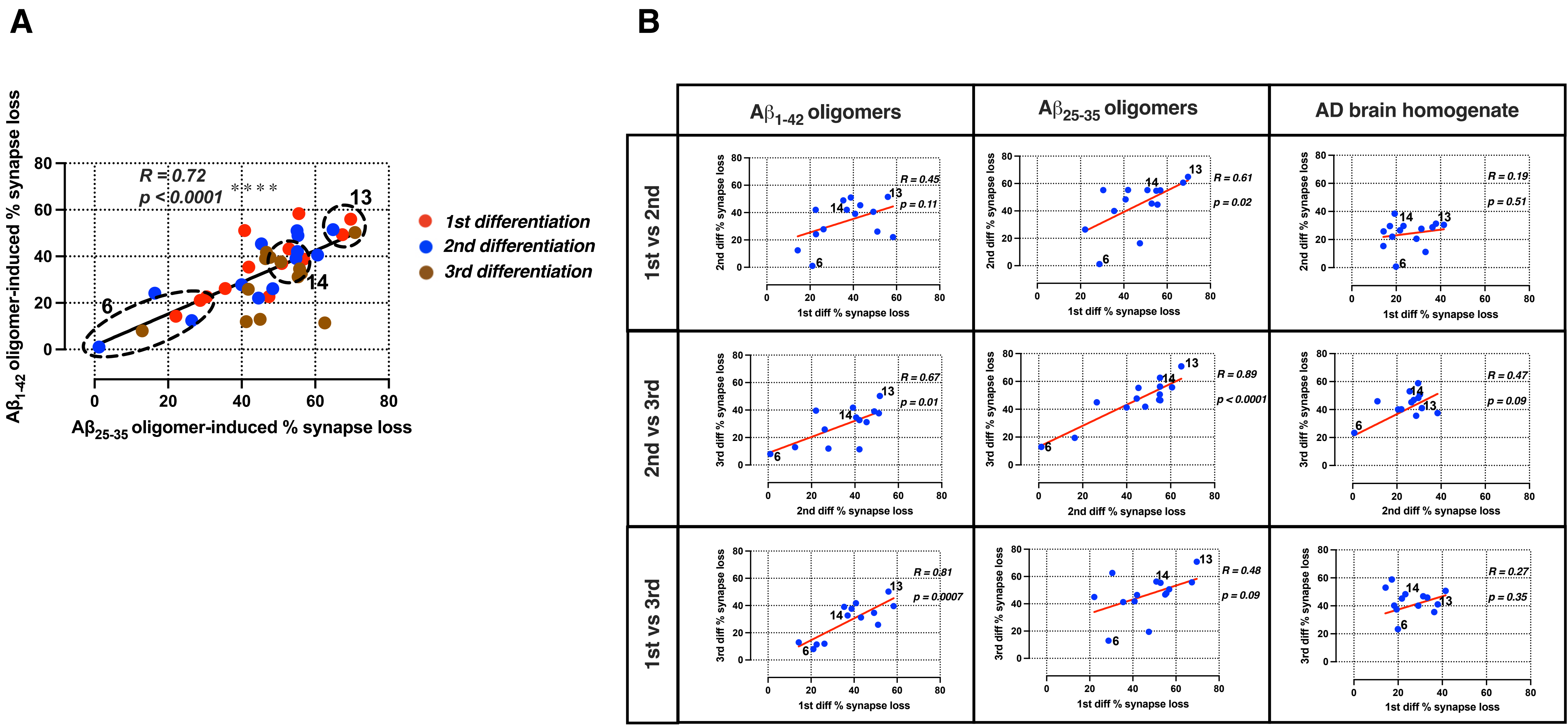

Supplementary Fig. 8

Supplementary Figure 8: Good reproducibility of the synapse loss data across neuronal differentiation repeats indicates cell-autonomous vulnerability to Aβ insults.

(A) Pairwise comparison on the degrees of synapse loss caused by either $A\beta_{1\text{-}42}$ or $A\beta_{25\text{-}35}$ oligomers. The zones where the same three selected patient lines from Fig. 2 can be found are circled in the graphs.

(B) Breakdown of individual pairwise comparisons on the degrees of synapse loss between differentiation repeats summarised in Fig 3. Each row denotes the two differentiation repeats in question and each column denotes the Aβ insult used to induce synapse loss.

Pearson's coefficient of correlation and its *p*-value are reported for statistical analysis.

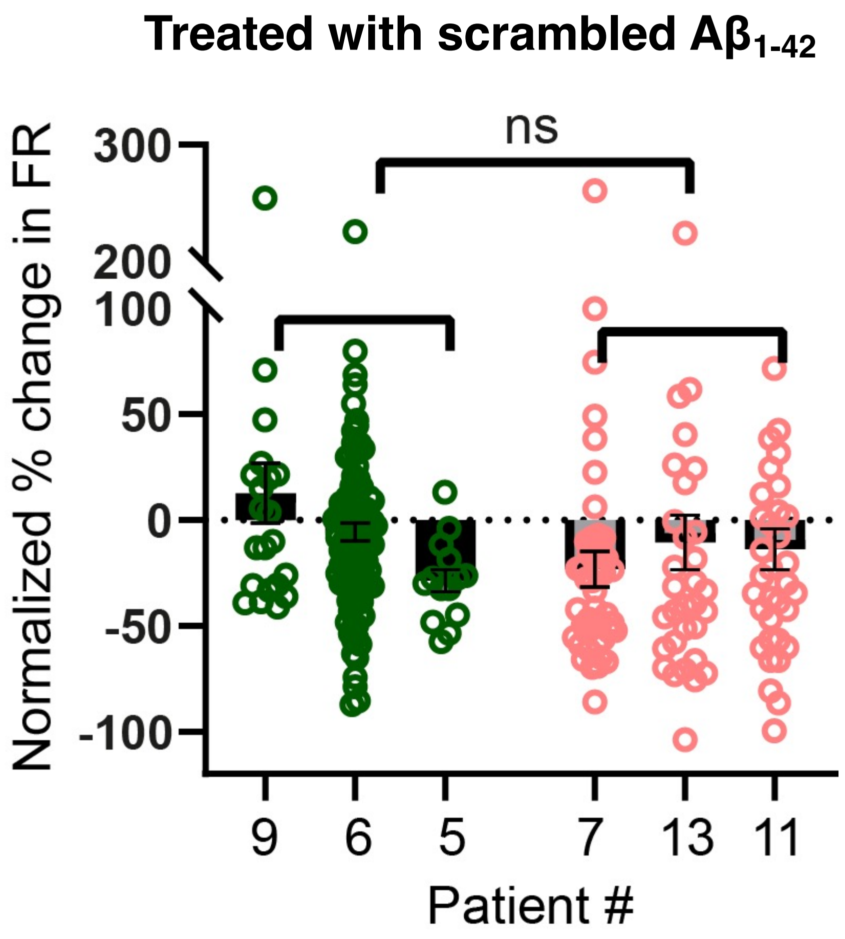

Supplementary Fig. 9

Supplementary Figure 9: Scrambled Aβ_{1-42} treatment did not cause any electrophysiological changes measured by MEA.

Comparison of the resilient group (Patients #9, #6 and #5; green) and vulnerable group (Patients #7, #13 and #11; red) neuronal response in their firing rate (FR) to scrambled Aβ_{1-42} 10 µM on the second day of incubation. Each datapoint represents an electrode recording. *n* = 22 (#9), 104 (#6), 14 (#5), 46 (#7), 33 (#13), and 35 (#11). Percentage change from baseline was normalised against changes of untreated media control. Mean ± SEM; Welch's *t*-test was used for statistical analysis.

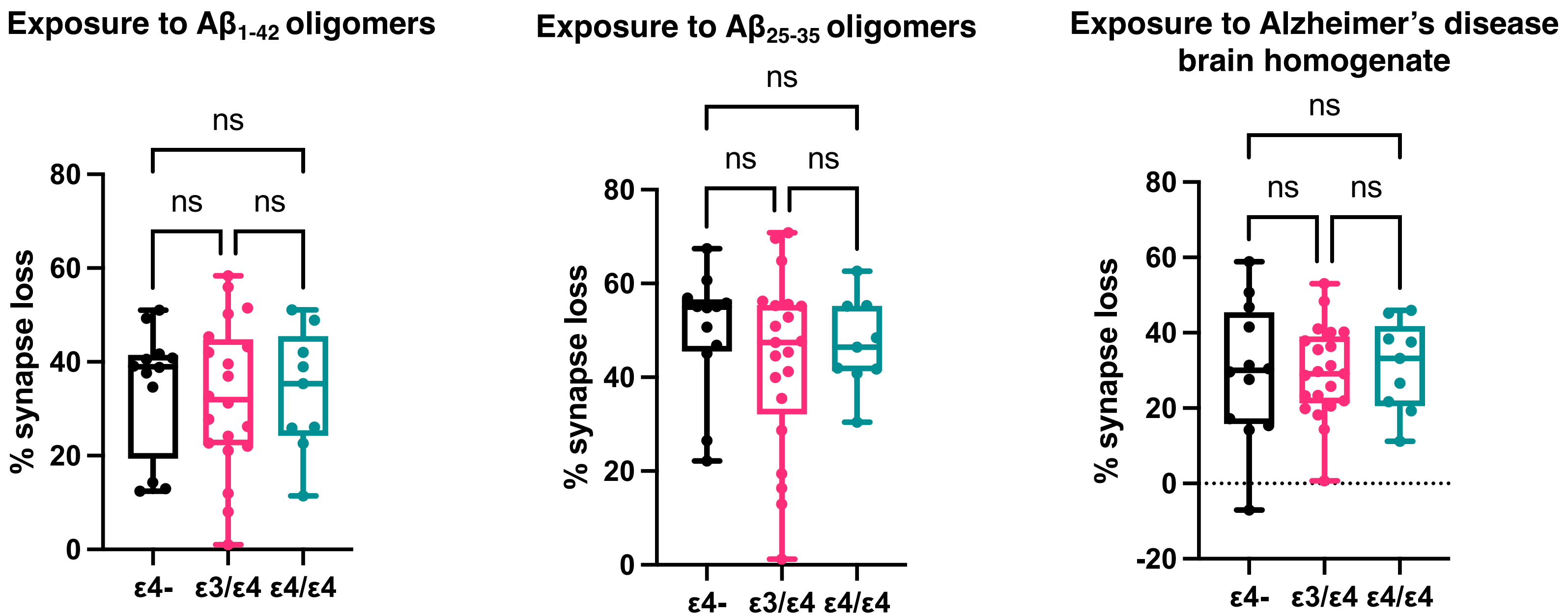

Supplementary Fig. 10

Supplementary Figure 10: *APOE* genotypes could not differentiate the synaptic vulnerability to extrinsic Aβ insults *in vitro*.

Box plots (centre line, median; box limits, interquartile range; whiskers, data range; points, all data points) showing the percentage of synapse loss caused by $A\beta_{1-42}$ oligomers, $A\beta_{25-35}$ oligomers and Alzheimer's disease brain homogenate with patients distinguished by their *APOE* variant genotypes. n = 12 (ε4-), 20 (ε3/ε4) and 9 (ε4/ε4) independent neuronal differentiation repeats per patient line. Kruskal-Wallis test was used for statistical analysis.

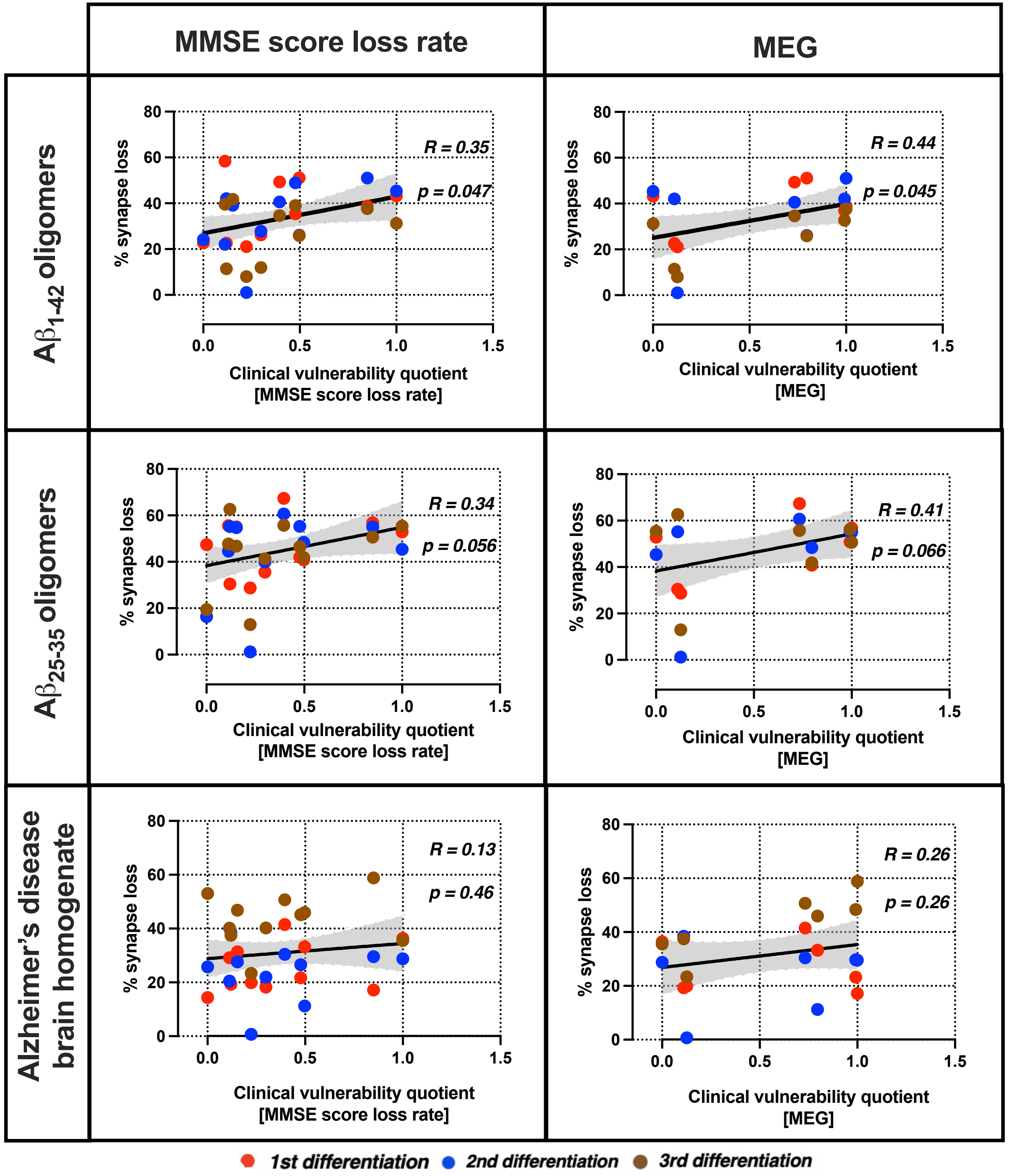

Supplementary Fig. 11

Supplementary Figure 11: Correlations between Aβ-driven synapse loss *in vitro* and clinical vulnerability to Aβ burden *in vivo*, as in Fig. 4, but without the familial Alzheimer's disease line (Patient #5).

Pairwise comparisons between the percentage of synapse loss and clinical vulnerability quotients. Each row denotes the type of extrinsic Aβ insult used to induce synapse loss and each column denotes the selected clinical outcomes which have been corrected for $A\beta_{1-42}$ concentration in the CSF (MMSE score loss rate) or amyloid PET SUVR (MEG). Error band: 95% CI. $n = 32$ ($A\beta_{1-42}$ - MMSE score loss rate), 33 ($A\beta_{25-35}$ and Alzheimer's disease brain homogenate – MMSE score loss rate) and 21 (all MEG) independent neuronal differentiation repeats per patient line. Pearson's coefficient of correlation and its *p*-value were reported for statistical analysis.
